# Exploring the Potential of Extracts from *Sloanea medusula* and *S. calva*: Formulating Two Skincare Gels with Antioxidant, Sun Protective Factor, and Anti-*Candida albicans* Activities

**DOI:** 10.3390/ph16070990

**Published:** 2023-07-11

**Authors:** Patricia Quintero-Rincón, Ana C. Mesa-Arango, Oscar A. Flórez-Acosta, Carolina Zapata-Zapata, Elena E. Stashenko, Nayive Pino-Benítez

**Affiliations:** 1 Natural Products Group, Technological University of Chocó, Quibdó 270002, Colombia; patriciaquintero@gmail.com; 2 Research Group Design and Formulation of Medicines, Cosmetics, and Related, Faculty of Pharmaceutical and Food Sciences, University of Antioquia, Medellín 050010, Colombia; oscar.florez@udea.edu.co; 3 Academic Group of Clinical Epidemiology, Faculty of Medicine, University of Antioquia, Medellín 050010, Colombia; ana.mesa@udea.edu.co (A.C.M.-A.); carolina.zapataz@udea.edu.co (C.Z.-Z.); 4 Center for Chromatography and Mass Spectrometry, CROM-MASS, CIBIMOL-CENIVAM, Industrial University of Santander, Bucaramanga 680002, Colombia; elena@tucan.uis.edu.co

**Keywords:** biodiversity, sustainability uses, plant-extract-based gels, *Sloanea* species, characterization, sun protective factor, skincare, *Candida albicans*, rheology

## Abstract

*Sloanea* is a plant genus, native to tropical regions, used in medicinal practices for its anti-inflammatory properties. This study aimed to determine the antioxidant activity, sun protective factor (SPF), and antifungal of extracts obtained from two species of *Sloanea* and to develop extract-based gels with antioxidants, photoprotective, and anti-*Candida albicans* effects. Ethanolic extracts from *S. medusula* and *S. calva* collected in Chocó, Colombia, were used for antioxidant activity and SPF determination using the DPPH assay and the Mansur equation, respectively. Extracts were characterized using HPLC-MS and used to prepare the gels. The viscosity of the extract-based gels was evaluated using an MCR92 rheometer. In addition, the anti-*Candida* activity of extracts against five yeasts and anti-*C. albicans* of gels were evaluated following the Clinical and Laboratory Standards Institute M27, 4th Edition. High DPPH radical scavenging activity (42.4% and 44.7%) and a high SPF value (32.5 and 35.4) were obtained for the extracts of *S. medusula* and *S. calva*, respectively. Similarly, extract-based gels showed significant DPPH radical scavenging activity of 54.5% and 53.0% and maximum SPF values of 60 and 57. Extract from *S. medusula* showed an important antifungal activity against *C. albicans* (minimal inhibitory concentration (MIC) of 2 µg/mL). In contrast, *S. calva* extract was active against *C. krusei*, *C. albicans* (MIC of 2 µg/mL) and *C. tropicalis* (MIC of 4 µg/mL). *Sloanea medusula* gel (0.15%) exhibited an important *C. albicans* growth inhibition (98%), while with *S. calva* gel (0.3%) growth inhibition was slightly lower (76%). Polyphenolic and triterpenoid compounds were tentatively identified for *S. medusula* and *S. calva*, respectively. Both extracts can be considered promising sources for developing photoprotective gels to treat skin infections caused by *C. albicans*.

## 1. Introduction

Throughout history, plant extracts have played a significant role in medicinal and cosmetic formulations [1,2]. Unfortunately, these valuable natural ingredients have gradually been substituted with chemicals, which have had a negative global impact, especially on ecosystems [3] and skin microflora [4]. Today, diverse strategies are being implemented to mitigate this impact and promote enhanced environmental consciousness. One of these strategies involves promoting natural extracts rich in antioxidant compounds as active ingredients as these represent a sustainable and eco-friendly alternative for pharmaceutical and cosmetic industries [5], especially for skincare product development [6], including sunscreens [7,8] and antimicrobial agents [9,10].

The Earth harbors a vast variety of ecosystems and provides the necessary conditions for a diverse range of plant species. Although extensive research has been conducted on the phytochemistry and biological activities of many of these species, it becomes apparent that numerous plants have not received the attention they truly deserve. This situation is particularly evident in the Colombian Chocó, a region located on the Pacific, recognized for its hyper-diverse flora that remain largely unexplored [11]. An example of these plants and the interest for our research is the genus *Sloanea*.

*Sloanea* is a plant genus native to tropical regions that comprises shrubs and tall trees, commonly called “achiotillo” by the local communities in Chocó. Plants within this genus have been used in traditional medicine to treat malaria, fever, and inflammation [12]. The phytochemistry of two species (*S. rhodantha* and *S. zuliaensis*) has been studied, revealing the presence of polyphenol compounds with antioxidant, anti-inflammatory, and antimicrobial properties, such as gallic acid and galloylquinic acid derivatives [13]. Additionally, triterpenoid compounds with cytotoxic properties against human cancer cells, such as cucurbitacin derivatives, have been identified [14]. This suggests that genus *Sloanea* could be an important source of compounds for skincare, protecting the skin exposed from damage caused by UV radiation and the treatment of fungal skin infections, e.g., cutaneous candidiasis [15]. *Sloanea* extracts could provide a sustainable alternative to chemical ingredients in personal care and beauty products.

Cutaneous candidiasis is a common fungal infection caused by *Candida* species, among them *C. albicans* [16]. This infection affects the skin, nails, and mucous membranes, resulting from an overgrowth of yeast on the skin [16], which leads to irritation, redness, itching, and flaking in the affected area [17]. Currently, treatment options include single-drug therapy and combinations of antimicrobials and corticosteroids [18] applying topical antifungal agents through creams, lotions, shampoos, or powders, or administering oral medications in severe cases [18,19]. However, there are major concerns regarding increasing antifungal resistance and relapses in treating *C. albicans* infections. New treatments for cutaneous candidiasis are being explored, such as nutritional products containing prebiotics and/or probiotics to restore healthy microbial flora of the skin and prevent fungal overgrowth [20,21]. New antifungal therapies are also being studied, including the use of nanoparticles [22] and plant-based topical formulations [23].

Plant-extract-based gels are crucial due to the health benefits provided by the bioactive compounds found in plants [23,24], mainly polyphenols with antioxidant and antimicrobial properties [25]. This has led to green consumption patterns, which are reflected in the growing demand for natural-ingredients-based products that offer cosmetic properties [26]. Additionally, they can be sustainably produced and formulated with environmentally friendly techniques [27]. Overall, plant-extract-based gels are a convenient and effective way to deliver bioactive compounds, making them an important option for topical formulations [28].

This study aimed to explore the antioxidant, photoprotective, and anti-fungal potential of ethanolic extracts obtained from *S. medusula* and *S. calva*, as well as to develop and characterize two gels based on extracts with very high SPF, antioxidant potential, and anti-*Candida albicans* activity, evaluated *in vitro*, to promote the sustainable use of wild plants in the Colombian Chocó for skincare.

## 2. Results

### 2.1. Characterization of the Extracts

#### 2.1.1. Chemical Composition of Ethanolic Extracts from *Sloanea* Species Leaves

Characterization by HPLC-MS/MS led to the tentative identification of seven compounds in the ethanolic extracts evaluated using electrospray ionization. Supplementary Figure S1 shows the extracted positive and negative ion chromatograms of the compounds present in the crude extracts.

Table 1, Table 2 and Supplementary Table S1 summarize the relevant data from the HPLC-MS analysis of the ethanolic extract of *S. medusula* and *S. calva*, respectively. The identified tentative molecules, possible fragment ions, neutral losses, and mass spectra are shown in Supplementary Figures S2–S8.

#### 2.1.2. Evaluation of the Antioxidant Activity of *S. medusula* and *S. calva* Extracts

The DPPH radical scavenging activity of the ethanolic extracts obtained from *S. medusula* and *S. calva* was evaluated using the DPPH assay at a concentration of 0.7 mg/mL. The results were expressed as the percentage of DPPH radical scavenging activity and Trolox equivalent antioxidant capacity (TEAC) (Table 3). As shown in Table 3, the extract of *S. medusula* showed significant radical scavenging activity (42.4%); however, the extract of *S. calva* showed slightly higher activity (44.7%). Moreover, the anti-radical efficacy expressed as TEAC showed values of 1.1 and 2.7 mM Eq. Trolox/100 g sample weight for the extracts of *S. medusula* and *S. calva*, respectively.

#### 2.1.3. Determination of *in vitro* Sun Protective Factor of *Sloanea* Extracts

The determination of SPF *in vitro* for the two *Sloanea* species evaluated is shown in Table 4. SPF values were evaluated at three concentrations, which ranged from 0.25 to 0.75 mg/mL, following the increasing order indicated below, *S. medusula* < *S. calva*. According to these results, at the concentration of 0.75 mg/mL, both evaluated extracts provide high protection against UVB radiation: *S. medusula* (SPF 32.5) and *S. calva* (SPF 35.4).

Additionally, as a complement to SPF, three photoprotective parameters were evaluated following the methodology employed by Caballero-Gallardo et al. [29]. Values of the UVA/UVB ratio, the transmission of erythema (%), and the transmission of pigmentation (%) showed the photoprotective potential of both extracts. Results are shown in Supplementary Table S2.

#### 2.1.4. Evaluation of the Anti-*Candida* Activity of *Sloanea* Extracts

*Sloanea medusula* and *S. calva* were evaluated at 10 concentrations ranging from 0.5 to 256 μg/mL to determine their MICs against *C. krusei* ATCC 6258 (L6), *C. auris* Ca17 (L25), *C. tropicalis* ATCC 200956, *C. glabrata* LMDM 34 (L7), and *C. albicans* ATTC 10231. The results, expressed as the geometric mean of the minimum inhibitory concentration (GM-MIC), are shown in Table 5. It can be observed that these yeasts have different sensibility profiles against two extracts evaluated, anti-*C. albicans* being potentially the most remarkable with an MIC of 2.0 μg/mL for both extracts, followed by *S. calva* against *C. krusei* (MIC of 2.0 μg/mL), *S. calva* against *C. tropicalis* (MIC of 4.0 μg/mL), and *S. medusula* against *C. auris* (MIC of 16.0 μg/mL). In addition, *C. krusei* ATCC 6258 (L6) was used as a control strain, and its susceptibility was evaluated using itraconazole (ITC) and amphotericin B (AMB), which showed MIC values of 0.125 μg/mL and 0.630 μg/mL, respectively.

### 2.2. Characterization of Sloanea-Extract-Based Gels

#### 2.2.1. Physicochemical Characterization of the Gels

##### Organoleptic Properties of Gels

**Figure 1:**
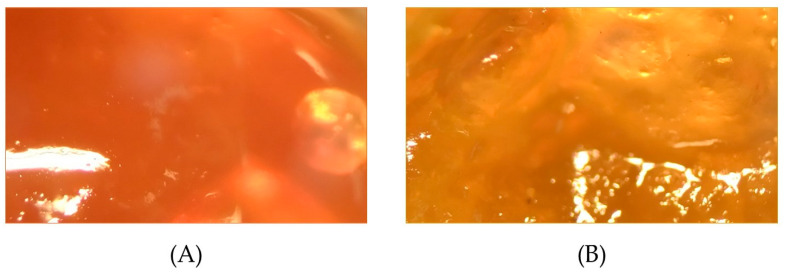
*Sloanea*-extract-based gels: (**A**) *Sloanea medusula*-based gel. (**B**) *Sloanea calva*-based gel.

Both the *S. medusula* (0.3%) and the *S. calva* (0.3%) gels showed a homogeneous appearance, without signs of lumps or precipitation. The *S. medusula* gel exhibited a terracotta color (Figure 1A) and a characteristic aroma of pleasant intensity, reminiscent of burnt grass, while the *S. calva* gel showed an ochre color (Figure 1B) and a slightly mentholated odor. The coloration of each gel was provided by the same plant extracts.

##### Rheology and Extensibility of *Sloanea*-Extract-Based Gels

Viscosity is frequently employed to analyze the characteristics of a formulation and ensure optimal distribution. It also enables formulators to enhance the efficacy of personal care products because this can be adjusted according to specific application needs [30].

**Figure 2:**
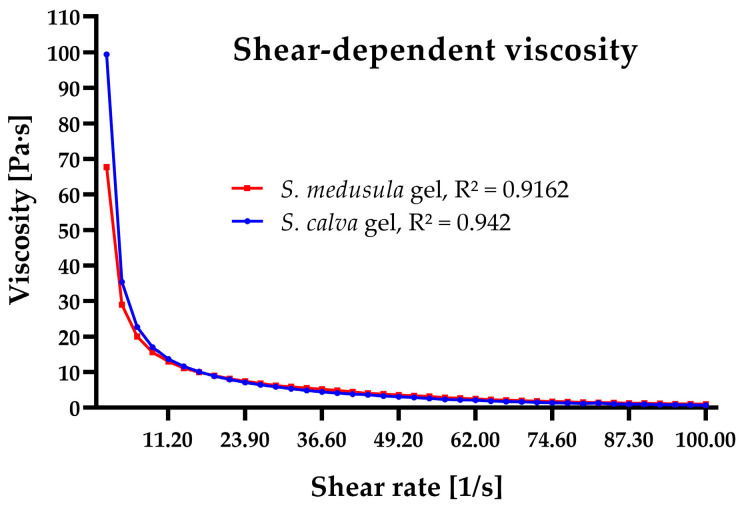
Rheological analysis showing flow behavior of *Sloanea*-extract-based gels.

In this study, the viscosity (Pa·s) of the gels was analyzed as a function of the shear rate (1/s) to monitor the stability of the formulation and define its rheological behavior. Figure 2 shows that the limiting low-shear viscosity of *S. calva* gel is higher in comparison with *S. medusula* gel. This is due to a higher concentration of the rheologic modifier (Carbopol^®^ Ultrez 21) in the *S. calva* formulation (Section 4.7). It is observed that the shear viscosity of the formulations decreases with increasing shear rate after a sufficiently large rate is attained. The descending part of the flow curve confirmed that both formulations exhibited non-Newtonian viscosity, i.e., pseudoplastic behavior [31].

The measurement of the extensibility area provides information about the deformation threshold of the formulations [32]. The extensibility area was measured by observing the increase in the surface of the gels under progressively increasing pressures (0.5958 g, 1.1624 g, and 4.2465 g) at 1 min intervals. This analysis resulted in the determination of extensibility area values, which provide valuable information about the capacity of *S. medusula*- and *S. calva*-based gels to stretch over the skin surface without experiencing breakage. Under these conditions, the *S. medusula*-based gel exhibited an extensibility area of 10.78 ± 2.5 cm^2^, showing a comparable elasticity or flexibility to the *S. calva*-based gel with an extensibility area of 10.56 ± 3.1 cm^2^.

#### 2.2.2. Efficacy Studies

##### Evaluation of the Antioxidant Activity of Extract-Based Gels by DPPH Assay

The results of antioxidant activity expressed as DPPH radical scavenging activity (%) and TEAC of gels based on *S. medusula* and *S. calva* extracts, respectively, are shown in Table 6. Antioxidant activity values were obtained at a concentration of 0.3 mg/mL. It is observed that the *S. medusula* gel exhibits the highest antioxidant capacity (54.5%, 22.9 mM Eq. Trolox/100 g of sample weight) compared to the *S. calva*-extract-based gel (53%, 20.3 mM Eq. Trolox/100 g sample weight).

##### Determination of *in vitro* Sun Protective Factor of Extract-Based Gels

The results of the determination of SPF for the two gels based on *Sloanea* extracts (0.3%) are shown in Table 7. To evaluate the photoprotective potential of the two formulations, a 1:1 ratio of gel and ethanol was prepared. The photoprotection indices obtained were FPS 60.0 and FPS 57.3 for the gels based on *S. medusula* and *S. calva*, respectively. The same methodology was used to evaluate the effectiveness of commercial sunscreen.

In addition to SPF, the UVA/UVB ratio, the transmission of erythema (%), and the transmission of pigmentation (%) were calculated following the criteria used in Section 2.1.3. Results are shown in Supplementary Table S2.

##### Evaluation of the Anti-*C. albicans* Activity of Extract-Based Gels

**Figure 3:**
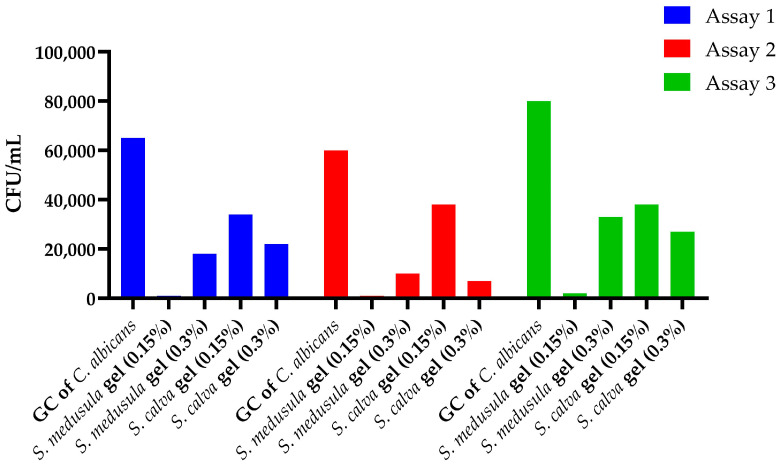
Determination of antifungal activity in *Sloanea*-extract-based gels by colony-forming units assay.

Two final concentrations (0.15% and 0.3%) of each gel were evaluated following the same protocol used to determine the MIC of *Sloanea* extracts against *Candida* species using 96-wheel plates. The experimental design is shown in Supplementary Figure S9. Then, three independent assays of colony-forming units were developed (Figure 3). The results of the inhibitory activity against *C. albicans* ATCC 10231 were expressed as the geometric mean of colony-forming units per milliliter (GM-CFU/mL) and percentage of yeast growth inhibition (Table 8).

The gel based on *S. medusula* showed the highest activity, with a 98% inhibition of *C. albicans* growth at a final concentration of 0.15%. Another notable result was obtained with the gel based on *S. calva*, which achieved a 76% inhibition of yeast growth at a concentration of 0.3%.

**Figure 4:**
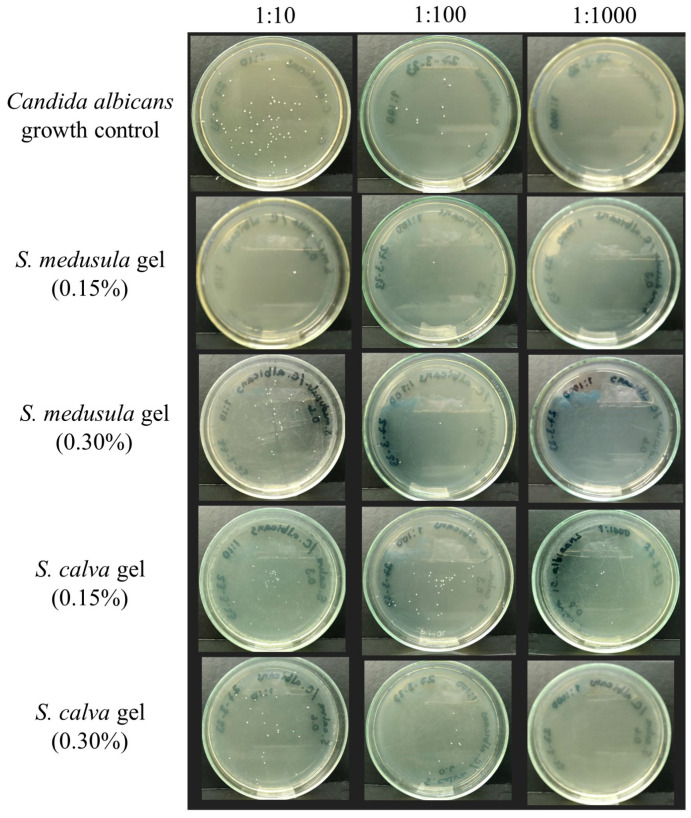
Evaluation of anti-*C. albicans* activity in *Sloanea*-extract-based gels by CFU assay.

Figure 4 corresponds to the results obtained in one of the assays developed for the determination of the percentage of inhibition of yeast growth in the formulations. Each white spot corresponds to a colony or colony-forming unit.

### 2.3. Statistical Analysis

The results were expressed as the mean ± standard error of the mean. The experimental data were collected at least in triplicate for each activity. The comparisons of results between the different groups were performed statistically using the paired *t*-test. The statistical significance was set at *p* < 0.05. GraphPad Prism 8.0 (GraphPad Software, San Diego, CA, USA) was used to show the flow behavior of *Sloanea*-extract-based gels in rheology analysis.

## 3. Discussion

*Sloanea* is a genus of shrubs and trees in the family Elaeocarpaceae, mainly distributed in tropical regions [33], which comprises approximately 150 species [34]. *Sloanea chocoana* is an endemic species of the Chocó Department in Colombia, which is found in the central rainforest of this region [35].

**Figure 5:**
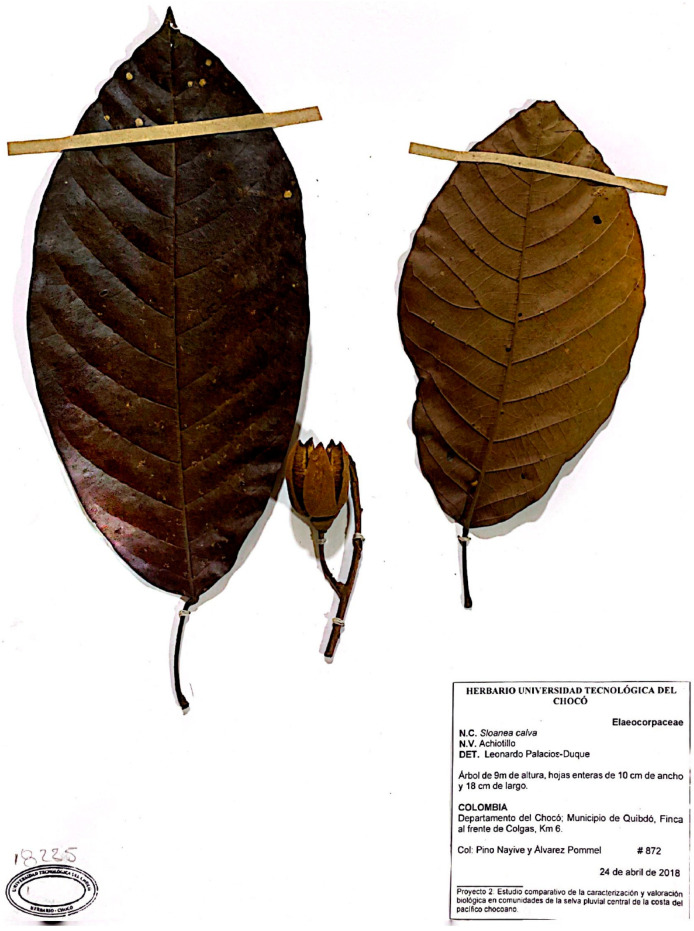
Botanical specimen of *Sloanea calva* Palacios-Duque & Fern. Alonso *sp. nov*.

*Sloanea calva* Palacios-Duque & Fern. Alonso *sp. nov.* (Figure 5) is a tree that grows from 0 to 135 m.a.s.l, ranging from 15 to 40 (52) m in height, and can be found in both primary and secondary forests in the Pacific and Central regions of Chocó. It is known as “Pulgo” in the municipality of Nuquí (Pacific coast) and as “Táparo” in the village of Salero, municipality of Unión Panamericana [36].

**Figure 6:**
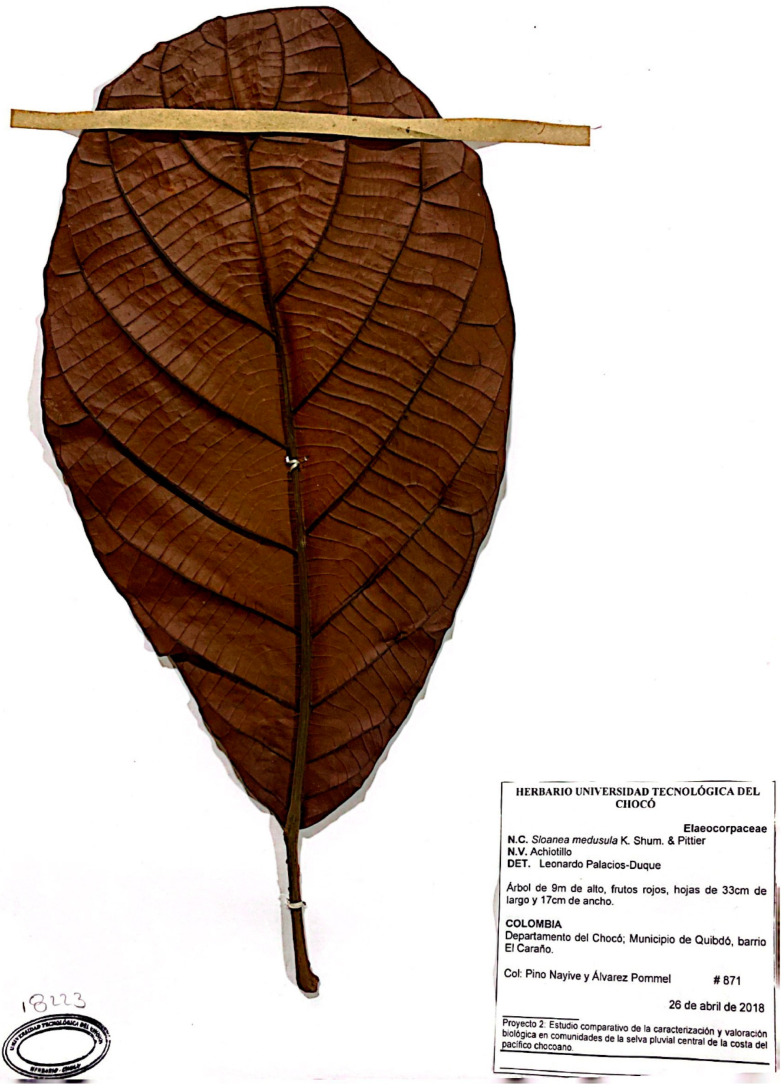
Botanical specimen of *S. medusula* K. Schum. & Pittier.

*S. medusula* K. Schum. & Pittier (Figure 6) is a tree that can reach heights between 12 and 35 m and is typically found at elevations ranging from 0 to 500 m.a.s.l. It has a broad distribution in the lowland areas of both the Caribbean and Pacific slopes, ranging from the central Pacific to the Osa Peninsula. In Nicaragua and Costa Rica, it is known as “Alma negra”, “Peine de mico”, and “Mano de león” [37].

Some species of *Sloanea* have traditionally been used in medicinal practices to treat ailments related to infectious and inflammatory processes [12], and their wood is often used to make axe handles, as well as for fuel or firewood [34].

Studies on the chemical characterization of two *Sloanea* species have revealed the presence of polyphenols and triterpenoids, including gallic acid, 3,5-di-O-galloylquinic acid, 1,6-di-O-galloyl glucopyranoside, 3,4,5-tri-O-galloylquinic acid, 1,2,3,6-tetra-O-galloyl glucopyranoside, 3,4,5-trimethoxyphenyl-(6’-O-galloyl)-O-b-D-glucopyranoside, 2-deoxycucurbitacin D, cucurbitacin D, and 25-acetylcucurbitacin F identified in *S. rhodantha* (Baker) Capuron var. rhodantha collected from the Madagascar rain forest and *S. zuliaensis* from Panama, respectively [13,14].

**Figure 7:**
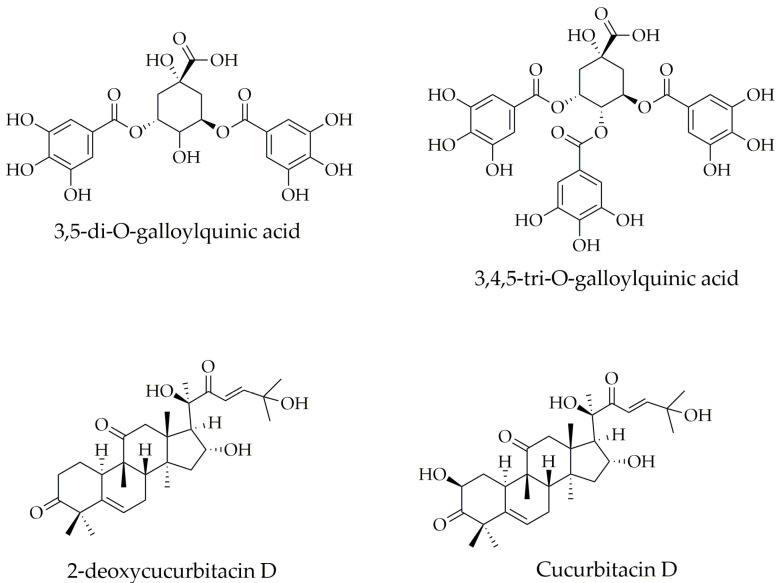
Some chemical structures of polyphenols and triterpenoids identified in *Sloanea* species.

The compounds identified in *Sloanea* species have shown interesting biological properties, which include the antifungal and antiplasmodial activity exhibited by some galloylquinic acid derivatives [13,38], and cytotoxic activity against human cancer cells, including breast (MCF-7), lung (H-460), and central nervous system (SF-268) exhibited by cucurbitacin analogs [14]. The chemical structures of some polyphenol and triterpenoid compounds identified in *Sloanea* species are shown in Figure 7.

In our study, the HPLC-MS analysis of the two extracts led to the tentative identification of seven compounds, including glycolic acid 4-hydroxy-3,5-di-t-butylbenzyl ester (identified in both *Sloanea* species), *α*-sorinin and two hydrolyzable tannins (granatin B and geraniin) for *S. medusula*, and three pentacyclic triterpenoids (uralenic acid, asiatic acid, and asiatic acid triacetate) for *S. calva*.

The peak at T_R_ 9.882 and *m*/*z* 293 was identified as the ion [M–H] of glycolic acid 4-hydroxy-3,5-di-t-butylbenzyl ester (C_17_H_26_O_4_). The fragment ion analysis and neutral losses in the MS-MS spectrum suggest the loss of a glycolic acid unit (76 Da) and 2-methyl-propane unit (58 Da) resulting in peaks at *m*/*z* 219 (C_15_H_23_O) and *m*/*z* 236 (C_13_H_17_O_4_) (Supplementary Figure S2). According to our review, this compound did not yield results from previous studies of fragment ion analysis in the ESI-MS^2^ spectrum.

The peak at T_R_ 3.4207 and *m*/*z* 539.123 was identified as the ion [M–H] of *α*-sorinin (C_24_H_28_O_14_), a compound related to the antioxidant activity observed in naphthalenic compounds from *Rhamnus nakaharai* [39]. The fragment ion analysis suggests the loss of a glucose unit and deoxyribopyranose unit for a total neutral loss of 314 Da, resulting in the peak at *m*/*z* 227 (C_13_H_7_O_4_) (Supplementary Figure S3). The molecular ion is observed at *m*/*z* 313 (C_11_H_21_O_10_) as a result of a neutral loss of the aglycone unit (226 Da). Finally, a total neutral loss of 152 Da corresponding to the deoxyribopyranose unit and water unit, led to fragment ion at *m*/*z* 387. This compound did not yield results from previous studies of fragment ion analysis in the ESI-MS^2^ spectrum.

The peak eluted at T_R_ 3.2289 and *m*/*z* 476.033, integrated for two compounds. This was identified as the [M–H] ion of ellagitannins corresponding to the formula C_41_H_28_O_27_ (tentative annotations: geraniin and granatin B). Ellagitannins are high molecular weight plant polyphenols found in woody and non-woody plants, which perform plant defense functions and are natural antioxidants that have demonstrated benefits for human and animal health due to their antimicrobial and antiparasitic properties [40]. The fragment ion analysis suggests the neutral loss of 782 Da and 651 Da, resulting in two peaks at *m*/*z* 169 (C_7_H_5_O_5_) and *m*/*z* 300 (C_14_H_5_O_8_) that correspond to the galloyl group (molecular ion) and a possible ellagic acid ion (Supplementary Figures S4 and S5), respectively. In the literature, ESI-MS analysis of geraniin (positive ion mode) has shown fragment ions at *m*/*z* 951, 554, 446, and 247 [41], and granatin B (negative ions mode) at *m*/*z* 951, 783, 605, and 300 [42].

The peak at T_R_ 8.044 and *m*/*z* 471.346886 was tentatively identified as the ion [M+H] of Uralenic acid or glycyrrhetinic acid (C_30_H_46_O_4_) (Supplementary Figure S6), a pentacyclic triterpenoid of oleanane-skeleton with remarkable antioxidant and antitumoral effects [43]. The fragments corresponding to ring breakage showed peaks at *m*/*z* 205 (C_15_H_25_), *m*/*z* 219 (C_15_H_23_O), and *m*/*z* 223 (C_14_H_23_O_2_). A total neutral loss of 64 Da, associated with one formic acid unit and one water unit, leads to a peak at *m*/*z* 407 (C_29_H_43_O). While at *m*/*z* 425 (molecular ion, C_29_H_44_O_2_), the neutral loss was associated with one formic acid unit (46 Da). ESI-MS analysis reported by other authors showed peaks at *m*/*z* 493, 471, and 177 [44].

The peak at T_R_ 5.953 and *m*/*z* 489.357451 corresponds to ions [M+H] of asiatic acid (C_30_H_48_O_5_). Asiatic acid is a pentacyclic triterpenoid of ursane-type with anti-inflammatory, antitumor, and antidiabetic properties [45]. Analysis of the mass spectrum of asiatic acid led to the identification of fragment ions at *m*/*z* 187 (C_10_H_35_O_2_), *m*/*z* 205 (molecular ion, C_15_H_25_), and *m*/*z* 223 (C_14_H_23_O_2_) generated by ring breakage. The total neutral loss of 82 Da associated with one unit of formic acid and two units of water generated a fragment ion at *m*/*z* 407 (C_29_H_43_O). Additionally, two fragment ions were observed at *m*/*z* 425 (C_29_H_45_O_2_) and *m*/*z* 453 (C_30_H_45_O_3_), due to neutral losses of formic acid and water (Supplementary Figure S7). The ESI-MS analysis in negative ion mode of asiatic acid has been previously reported, showing peaks at *m*/*z* 487 [46], *m*/*z* 688, 455, and 365 [47], and *m*/*z* 409 [48].

The last analyzed peak eluted at T_R_ 4.389 and *m*/*z* 615.38914 was tentatively identified as the [M+H] ion of asiatic acid triacetate (Supplementary Figure S8). Fragment ion analysis suggested three important peaks at *m*/*z* 219 (C_16_H_27_), *m*/*z* 407 (C_29_H_43_O), and *m*/*z* 453 (C_31_H_49_O_2_) generated by neutral losses of 396 Da (C_20_H_28_O_8_), 206 Da (three acetic acid unit and one ethylene unit) and 162 Da (two acetic acid units and one propene unit), respectively. Peaks at *m*/*z* 628, 612, and 605 were observed in the MS^2^ spectrum reported in the literature using ESI-MS in negative ion mode [47].

In general, identified compounds in HPLC-MS analysis have exhibited properties that benefit skin health, such as antimicrobial, antioxidant, anti-inflammatory, chemoprotective, cytotoxic, and wound-healing properties [49,50,51,52]. Therefore, these compounds may be responsible for the observed biological activities. These findings create an opportunity for bioprospecting and evaluating the potential use of extracts from other *Sloanea* species, active fractions, or compounds in the cosmetic and pharmaceutical industries.

Various studies have shown the potential of plant extracts in skincare product development, especially for cutaneous candidiasis treatment induced by *C. albicans* [53]. In addition, the protective effects against premature aging have been addressed, due mainly to the photoprotective and antioxidant properties of natural extracts [54]. Regarding formulations, gels have emerged as a promising alternative for skin infection treatments. Their use implies lower toxicity compared to oral or intravenous antifungals, excellent chemical stability, and enhanced penetration in infected areas, making them effective delivery agents for multiple compounds [55]. The benefits of plant-extract-based gels have been observed in emulgels loaded with clove/cinnamon oils, which are an alternative for treating *C. albicans*-associated denture stomatitis [56], and in hydrogels integrating lemongrass-loaded nanosponges showing an enhanced antifungal effect with reduced irritation [57].

Currently, the photoprotective properties of extracts [58], essential oils [29], and secondary metabolites [59] are being explored, and the antifungal activity of these natural extracts [60] and compounds [61] has highlighted the potential of Colombian flora for the development of topical formulations.

In this study, ethanolic extracts obtained from *S. medusula* and *S. calva* collected in Chocó, Colombia, exhibited antioxidant effects, with values that are considered satisfactory for natural extracts [62]. This activity plays a fundamental role in the photoprotective and antifungal properties observed in natural extracts [63,64]. Results obtained showed that the extracts and *Sloanea*-extract-based gels offer high and maximum SPF values. Moreover, a noteworthy reduction in values *in vitro* of percentages of transmission of erythema and pigmentation (Supplementary Table S2) suggests the ability of these extracts and formulations to protect the skin exposed to sunlight from redness and dark spots, respectively. The results obtained with the evaluated extracts exceed those reported for *Holoptelea integrifolia* extract (Ulmacea), a species that shares traditional uses similar to those reported for the *Sloanea* species, including malaria, rheumatism, common fever, as well as other conditions related to rickets, leprosy, leucoderma, among others [65].

Our results demonstrate that extracts from *S. medusula* and *S. calva* exhibit significant antifungal properties, particularly against the yeast *C. albicans*, and these properties are preserved in the gels. However, the disparity observed in the gels made with *S. medusula* is noteworthy, where a concentration of 0.15% of the extract showed a superior inhibitory effect compared to the highest concentration evaluated. Concerning that, phenomena have been described in the literature in different species of *Candida* exposed to echinocandins (an antifungal whose target is the enzyme B-1,3 glucan synthase, responsible for the synthesis of glucans in the fungal wall). This phenomenon is known as a paradoxical effect. It is caused by exposure to high concentrations of echinocandins, which induce stress adaptation pathways by increasing cell wall chitin *in vitro* in response to the depletion of wall glycans and contributing to fungal growth [66]. This growth makes the interpretation of *in vitro* susceptibility tests difficult. However, its relevance *in vivo* has not been proven. Although our results do not allow us to elucidate what is happening, the increase in the growth of *C. albicans* at high concentrations of the *S. medusula* extract and its inhibition at low concentrations could be related to a similar effect.

The gels obtained showed pseudoplastic behavior and extensibility areas that lead to a favorable dispersion during its topical application, which favors the creation of a uniform film on the skin surface, contributing to the efficacy of the formulations [67]. However, a decreased antifungal efficacy of the *S. calva*-based gel was observed, which could be mainly attributed to the composition of the polymer within the gel. The literature suggests that the active ingredients in the gel, such as crude extracts, can strongly interact through the –OH, -COOH, and C-O-C groups with the polymer via hydrogen bonding [68]. This interaction results in higher viscosity and reduced release capacity of the active compounds. Therefore, adjusting the polymer composition within the formulation may lead to improved efficacy.

Although it is not common for topical treatments for infections caused by *C. albicans* to cause skin pigmentation, some treatments can cause irritation or dryness (e.g., Gentian violet, Nystatin, Miconazole, Ketoconazole, and Clotrimazole) and ulcers (e.g., Gentian violet), making the skin more sensitive to sunlight and potentially leading to dark spots [69].

It is important to note that using a formulation with both SPF and anti-*Candida albicans* activity can be highly beneficial. Sunscreen helps protect the skin from UV damage, which can avoid skin irritation and pigmentation issues. Incorporating an anti-*C. albicans* agent into the formulation can help address the root cause of the infection and prevent further occurrences.

Today, cutaneous candidiasis caused by *C. albicans* has emerged as a critical health concern, demanding immediate attention and concerted efforts toward continuous research and development of therapeutic options. An enhanced prevalence of cutaneous candidiasis becomes a substantial burden for healthcare systems and public health in general [18]. Hence, prioritizing and investing in ongoing research becomes pivotal to mitigate the adverse impact of this disease and enhance the quality of life for affected individuals.

Future directions of this study will lead to the optimization of both gels to enhance their antifungal properties. The optimization of viscosity and rheological behavior of the gels should be accompanied by pH measurement, which should be ≤5.5 for topical formulations [70], to preserve the integrity of the skin barrier [71]. This should be followed by *in vitro* tolerance studies, evaluation of genomic integrity, and *in vivo* studies. Furthermore, it would be interesting to study the underlying mechanisms related to the inhibitory effect on the growth of *C. albicans* using the gel based on *S. medusula* extract.

## 4. Materials and Methods

### 4.1. Materials

All available reagents and solvents were obtained from commercial suppliers and used without further purification. These were acquired from Fisher Scientific (Acetic acid, Waltham, MA, USA), Sigma-Aldrich Co. (Amphotericin B, Itraconazole, Sodium acetate, Sabouraud dextrose agar, and RPMI 1640-MOPS, St. Louise, MO, USA), Aldrich (Trolox, St. Louise, MO, USA), and Sigma Chemical Co. (DPPH radical; 1,1-diphenyl-2-picrylhydrazyl, St. Louise, MO, USA), Merck (Methanol, acetonitrile, and water, LC/MS grade, Rahway, NJ, USA), Lubrizol Corporation (Carbopol^®^ Ultrez 21 Polymer or Acrylates/C10-30 Alkyl acrylate crosspolymer, Wickliffe, OH, USA), Clariant (Sodium nipagin or Sodium methylparaben, Muttenz, Switzerland), ASES Chemical works (Triethanolamine 99%, Rajasthan, India), and Phylusa corporation (Glycerine, Pasig City, Philippines). Finally, 96-well microdilution plates were purchased from Corning^®^ (Sommerville, MA, USA) and Costar^®^ (Washington, DC, USA).

### 4.2. Plant Material Collection and Extraction

The plants under study were collected in Quibdó, Chocó Department, Colombia: Barrio El Caraño (*S. medusula*), and farm in front of Colgas, at Km 6 (*S. calva*). The specimens were deposited in the Herbarium of the Technological University of Chocó (Quibdó), along with their corresponding vouchers (voucher number in parentheses): *S. medusula* K.Schum. & Pittier, (871 Herbario CHOCÓ), and *S. calva* Pal.-Duque & Fern. Alonso (872 Herbario CHOCÓ). The scientific names of both species under study were determined by Ph.D. Leonardo Palacios-Duque, a specialist in *Sloanea* species and professor at the Technological University of Chocó.

For each specimen, the leaves were air-dried, followed by a grinding process using a ball mill. The ground plant material was macerated (no stirring) with 96% ethanol for 72 h. The solvent was filtered, and the residue was extracted two-fold. The resulting extracts were evaporated and dried using rotary evaporation until the crude extracts were obtained. The yield of each extract was 1.1% (*S. calva*) and 1.2% (*S. medusula*) relative to the fresh plant. The obtained extracts were labeled and stored at 4 °C until use.

The extraction was carried out using ethanol, as it is one of the most commonly used solvents in the traditional medicine practices of Chocó communities. This solvent facilitates the extraction of compounds with therapeutic properties of interest, such as flavonoids, tannins, phenolic compounds, and triterpenoids, among others [72].

### 4.3. HPLC-MS Analysis

The extracts were separately diluted in methanol. Each diluted sample was vigorously shaken to achieve homogenization. Subsequently, a filtering process was applied using a 0.22 μm pore size membrane, and the filtered samples were transferred to autosampler vials. The extract was separated and analyzed using a UHPLC (Dionex Ultimate 3000, Thermo Scientific, Waltham, MA, USA), coupled to an ESI q-QTOF (Impact 2, Bruker Daltonics), operated in positive and negative ion modes. Separation was carried out using a Luna C18 column, 4.6 × 150 mm, 3 μm particle size (Phenomenex, Torrance, CA, USA). The column temperature was maintained at 30 °C. The mobile phase consisted of water (A) and acetonitrile (B). The analysis started with 40:60 A: B, held for 5 min, followed by changing linearly up to 100% B in 5 min, then returned to 40:60 A: B in 5 min, and held until 25 min. The flow was 0.4 mL/min and the injection volume of 5 μL. The conditions for the mass detector were as follows: Capillary voltage +3.5 kV (negative ion) and +4.5 kV (positive ion), nitrogen gas temperature 220 °C, drying gas flow rate 8.0 L/min, and nebulizer gas pressure 1.8 Bar. MS/MS data acquisition mode was used to assist compound identification. The mass range in MS/MS experiments was set at *m*/*z* 50–1300. Global Natural Products Social Molecular Networking (GNPS; http://gnps.ucsd.edu accessed on 6 June 2023) was used for the analysis of MS/MS data [73].

### 4.4. DPPH Assay

The study assessed the hydrogen atom donating ability of plant extracts and gels by measuring the decolorization of a methanol solution of DPPH, which changes from violet/purple to shades of yellow in the presence of antioxidants [74]. The DPPH radical scavenging assay was performed following the method reported in the literature [75], with some modifications. Briefly, a solution of DPPH 0.125 mM was prepared by dissolving 0.00197 g of the DPPH radical in 15 mL methanol, and the volume was adjusted with acetate buffer until the absorbance of the working solution was 1.0 ± 0.2 at 517 nm. The standard solution was prepared by dissolving 2 mg of Trolox (97% purity) in 10 mL of 80% methanol. The solution obtained had a deep purple color and was left in the refrigerator for 2 h for the absorbance to be stabilized. Then, a curve of Trolox standard solution was prepared in a concentration range from 0.08 mM to 2 mM. These solutions were vortexed and left in darkness and their absorbance was measured using a Varioskan™ LUX Multimode Microplate Reader (Thermo Fisher Scientific, Inc., Waltham, MA, USA) at λ_max_ of 517 nm. The calibration curve of absorbance (y) versus concentration (x) of DPPH is expressed by the following equation (y = −0.16x + 0.4792, R^2^ = 0.9973). Subsequently, 0.05 mL of extract solution (0.7 mg/mL) or gel (0.3 mg/mL) is added to 1.95 mL of the DPPH radical working solution. The mixture is carefully homogenized and kept in the dark for 30 min. Finally, 200 µL per triplicate is dispensed into a 96-well plate and the absorbance is measured at 570 nm. For the antioxidant assay, methanol was used as the blank.

The percentage of DPPH radical scavenging activity was calculated by the following equation:

(1) $$\mathrm{DPPH}\;\mathrm{radical}\;\mathrm{scavenging}\;\mathrm{activity}\;\left(\%\right)=\left[\frac{A_{0}-A_{1}}{A_{0}}\right]*100$$

where A_0_ is the absorbance of the control, and A_1_ is the absorbance of the *Sloanea* extracts/extracts-based gels.

Then, the results expressed as TEAC (Trolox equivalent antioxidant capacity) (µmolar/100 g sample weight) were calculated from a calibration curve obtained by linear regression, following the equation:

(2) $$\frac{µM\;\mathrm{Eq}.\;\mathrm{Trolox}}{100\;g\;\mathrm{sample}\;\mathrm{weight}}=\frac{M*\mathrm{FD}*V}{m}\;*100$$

where M is the molar concentration obtained from the calibration curve, FD is the dilution factor used to prepare the sample, V is the volume in liters in which the sample was initially prepared, and m is the weight in grams of the initial sample.

### 4.5. Evaluation of Sun Protection Factor of Sloanea Extracts and Sloanea-Extract-Based Gels

The SPF was determined by measuring the absorbance values between wavelengths of 290 to 320 nm (UVB spectrum) using an optical path of 5.0 cm in a Varioskan^™^ LUX Multimode Microplate Reader (Thermo Fisher Scientific, Inc., Waltham, MA, USA). The calculated SPF value was then applied to the Mansur equation [76]:

(3) $$\mathrm{SPF}=\mathrm{CF}\times \sum_{290\;\mathrm{nm}}^{320\;\mathrm{nm}}\mathrm{EE}\;\left(\lambda \right)\times I\left(\lambda \right)\times \mathrm{Abs}\;\left(\lambda \right)$$

In this equation, CF represents the correction factor (equal to 10), while EE(λ) × I(λ) are constants at each wavelength (Table 9) obtained from the correlation between the erythemal effect (EE) of radiation of wavelength λ and the solar intensity at the same wavelength (I) [77]. Additionally, Abs refers to the absorbance of the solution at wavelength λ [78]. The CF value was determined so that a standard sunscreen formulation containing 8% homosalate would have an SPF value of 4, measured by UV spectrometry [79]. SPF values between 2–15 indicate low protection against UV rays, values between 15–30 indicate moderate protection, values between 30–50 indicate high protection, and values greater than 50 indicate maximum protection against UV rays [29].

### 4.6. Evaluation of the Inhibitory Effects of Extracts and Extract-Based Gels on the Growth of Candida albicans

#### 4.6.1. Fungal Strain

The fungal strains used in this study were *C. krusei* ATCC 6258 (L6) (*Issatchenkia orientalis* ATCC 6258), *C. auris* Ca17 (L25), *C. tropicalis* ATCC 200956, *C. glabrata* LMDM 34 (L7), and *C. albicans* ATCC 10231. The activity of ITC and AMB against *C. krusei* ATCC 6258 was evaluated in all experiments as an antifungal susceptibility testing control following the Clinical and Laboratory Standards Institute M27, 4th Edition (CLSI standard M27, 4th Edition) [80], it was mandatory that the MIC values remained within the accepted range, i.e., MIC values of 0.125 µg/mL and 0.630 µg/mL for ITC and AMB, respectively [81]. All yeasts were cultured on Sabouraud Dextrose Agar for 24 h at 35 °C.

#### 4.6.2. Antifungal Susceptibility Testing

The antifungal susceptibility testing was performed according to the CLSI standard M27, 4th Edition [80]. Some modifications were applied to evaluate the antifungal effects of extracts. Briefly, stock solutions of extracts were prepared at 512 µg/mL, and an inoculum of 2.5 × 10^3^ CFU/mL of each yeast was prepared in RPMI 1640-MOPS. Then, 100 µL of each stock solution of extracts were added to 96-well microdilution plates to finally evaluate 10 two-fold dilutions ranging from 0.5 to 256 µg/mL, followed by 100 µL of each yeast inoculum (evaluated concentration of the inoculum was 1.25 × 10^3^ CFU/mL). The microdilution plates were again incubated at 35 °C for 24 h. The MICs were visually determined as the lowest concentration that produced visual inhibition compared to the growth control using a manual mirror viewer. The assays were performed at least three times in duplicate on different days, and the results were expressed as GM-MIC.

The antifungal susceptibility testing described to evaluate the antifungal effects of extracts was used to evaluate the susceptibility of *C. albicans* against the *Sloanea*-extract-based gels at two final concentrations (0.15% and 0.3%), including the gel base as a growth control (Supplementary Figure S9). To obtain results of the antifungal activity of the gels at a final concentration of 0.3%, formulations were prepared by doubling the concentration of the extract (i.e., 0.6% *w*/*w*) while maintaining the same concentrations of the other components in the formulation. An inoculum of 2.5 × 10^3^ CFU/mL of *C. albicans* was prepared in RPMI 1640-MOPS (2X) to evaluate the antifungal effects of the base gel and *Sloanea*-based gels. Gel (100 µL) was added to 96-well microdilution plates, followed by 100 µL of inoculum (the evaluated final concentration of the inoculum was 1.25 × 10^3^ CFU/mL). After 24 h of incubation at 35 °C, the colony-forming unit assay was performed to determine the CFU/mL, and the results were expressed as GM-CFU/mL and yeast growth inhibition (%). The CFU assay involved the preparation of 1:10, 1:100, and 1:1000 dilutions. To prepare 1:10 dilutions, one well per concentration was randomly chosen (Supplementary Figure S9). A volume of 10 µL was taken from the well and diluted in sterile deionized water (90 µL). Subsequently, 1:100 and 1:1000 dilutions were prepared. Once the dilutions were prepared, a volume of 10 µL was taken, which was transferred to the center of each plate and spread in the solid agar medium (Sabouraud dextrose agar) with the help of 4–5 glass beads for the purpose of allowing viable cells to form colonies under specific growth conditions. The beads were removed, and the plates were subjected to an incubation period at 35 °C. After 24 h, the colonies were counted, and the CFU/mL was calculated based on the 1:10 dilution factor.

### 4.7. Preparation of Sloanea-Extract-Based Gels, Rheology, and Extensibility

To prepare both gels, *Sloanea* extract was first dissolved in deionized water. This solution was then added to a mixture containing carbopol^®^ ultrez 21 polymers, sodium nipagin, triethanolamine, glycerine, and 100% deionized water q.s.p. (Table 10), which was prepared at 40 °C. The mixture was thoroughly dissolved. Additionally, a blank formulation (negative control) was prepared under the same conditions, excluding the incorporation of the extract.

During the preparation of the base formulation and optimization of the final formulations, differences were observed in the behavior of the base gel when it came into contact with the plant extract. First, the rheology modifier (Carbopol) in the base gel was adjusted until the standardization of the *S. medusula*-based gel. Then, to standardize the gel based on *S. calva*, it was necessary to increase the concentration of Carbopol until a formulation that offered rheological properties similar to those obtained with the first extract was achieved. These tests resulted in the concentrations shown in Table 9. The physicochemical behavior of the gels against evaluated natural extracts would be an interesting topic to address in future research.

The organoleptic properties of the gels based on *Sloanea* extracts were assessed by two individuals at different times. Images of gels were acquired using a stereomicroscope with a 2× objective (Zivot).

Rheological analysis was performed using an MCR92 rheometer (Anton-Paar^®^, Graz, Austria). The viscosity of the gels was run with 50 mm cone-and-plate to 25 ± 0.5 °C. Samples of the two gels were stored at room temperature. Apparent viscosity and rheological behavior were determined at 28 days. The graph of flow behavior was generated using GraphPad Prism 8.0.

The extensibility area of the gels was determined at a room temperature of 25 ± 0.5 °C using the methodology of Pérez-Bueno et al. [32], with minor modifications. Briefly, a known amount of the sample was placed at the center of a flat glass surface. Subsequently, another flat glass plate with a known weight (average: 0.5958 g) was placed on top of the sample. After waiting for 1 min, the extended radius of the sample was measured. The same measurement was repeated after adding two additional flat plates with known weights (average values: 1.1624 g and 4.2465 g, respectively). The measurements were performed in triplicate. Based on the three data points, a curve of radius vs. weight was plotted, and the slope expressed in cm/g was determined.

The slope of *S. medusula* gel was 2.917 cm/g for an average sample weight of 0.635 g, and the slope of *S. calva* gel was 2.095 cm/g for an average sample weight of 0.875 g.

The average radius was then calculated through the equation:

(4) $$\mathrm{Average}\;\mathrm{radius}\;\left(\mathrm{cm}\right)=\mathrm{slope}\times \mathrm{average}\;\mathrm{weight}$$

Finally, the extensibility area expressed in cm^2^ was calculated using the following equation:

(5) $$\mathrm{Extensibility}\;\mathrm{area}\;\left({\mathrm{cm}}^{2}\right)=\pi {\left(\mathrm{average}\;\mathrm{radius}\right)}^{2}$$

## 5. Conclusions

This study constitutes the first report on the chemical composition and biological activities of the ethanolic extracts obtained from *S. medusula* and *S. calva* leaves.

Chemical composition was studied using the HPLC-MS method. Two hydrolyzable tannins (granatin B and geraniin) were tentatively identified in *S. medusula* extract, while three pentacyclic triterpenoids (uralenic acid, asiatic acid, and asiatic acid triacetate) were tentatively identified in *S. calva* extract. These compounds are widely recognized as antioxidant, anticancer, and antimicrobial agents, suggesting a relation with the observed biological activities. 

Our results indicated that both extracts possess high antioxidant and photoprotective activity. In addition, promising antifungal activity was observed against different *Candida* species, although the susceptibility of the tested strains varied. MIC values of 2 μg/mL were obtained against *C. albicans*, a yeast species associated with cutaneous candidiasis. In summary, these results suggest the beneficial effects of *S. medusula* and *S. calva* extracts on the skin, and further research is recommended to determine the effects of these extracts on human health.

The gel based on *S. medusula* evaluated at a final concentration of 0.15%, offers suitable characteristics as an anti-*Candida albicans* agent. This makes it an attractive formulation and could constitute a promising alternative in cutaneous candidiasis treatment.

Future directions for this work include the *in vitro* tolerance studies, evaluating genomic integrity, and performing *in vivo* studies. Additionally, the study of underlying mechanisms behind the inhibitory effect on *C. albicans* growth using the gel containing *S. medusula* extract and the physicochemical behavior of gel based on *S. calva* extract are of particular interest.

## Figures and Tables

**Table 1:** HPLC–MS analysis of *S. medusula* extract.

No.	T_R_ (min)	Adduct	Estimated Mass (*m*/*z*)	Exact Mass [M] (g/mol)	Structural Formula	Error (ppm)	Tentative Annotation
1	9.882	M-H	293.17583	294.18367	C_17_H_26_O_4_	−2.8	Glycolic acid 4-hydroxy-3,5-di-t-butylbenzyl ester
2	3.4207	M-H	539.1230	540.14734	C_24_H_28_O_14_	2.5	*α*-Sorinin
3	3.2289	M-H	952.08289	952.08289	C_41_H_28_O_27_	2.1	Geraniin
			476.03300				
4	3.2289	M-H	952.08289	952.08289	C_41_H_28_O_27_	2.1	Granatin B
			476.03300				

**Table 2:** HPLC–MS analysis of *S. calva* extract.

No.	T_R_ (min)	Adduct	Estimated Mass (*m*/*z*)	Exact Mass [M] (g/mol)	Structural Formula	Error (ppm)	Tentative Annotation
1	9.882	M-H	293.17583	294.18367	C_17_H_26_O_4_	−2.8	Glycolic acid 4-hydroxy-3,5-di-t-butylbenzyl ester
2	8.044	M+H	471.346886	470.33905	C_30_H_46_O_4_	−0.1	Uralenic acid, glycyrrhetinic acid
3	5.953	M+H	489.357451	488.34961	C_30_H_48_O_5_	−0.0	Asiatic acid
4	4.389	M+H	615.389145	614.38131	C_36_H_54_O_8_	−0.1	Asiatic acid triacetate

**Table 3:** Results of DPPH assays of *Sloanea* extracts.

Extract	DPPH ^a^ Radical Scavenging Activity (%) MEAN ± SEM (*n* = 3)	TEAC ^b^ MEAN ± SEM (*n* = 3)
*S. medusula*	42.4 ± 0.0	1.1 ± 0.0
*S. calva*	44.7 ± 0.0	2.7 ± 0.0

^a^ DPPH (1,1-diphenyl-2-picrylhydrazyl). ^b^ TEAC (Trolox equivalent antioxidant capacity). Results were expressed as the mean ± standard error of the mean from three independent assays.

**Table 4:** Results of the determination of sun protective factor of extracts from *Sloanea* species.

Extract	SPF ^a^ MEAN ± SEM (*n* = 3)		
	0.25 mg/mL	0.5 mg/mL	0.75 mg/mL
*S. medusula*	16.9 ± 0.1 ^b^	26.7 ± 0.6 ^c^	32.5 ± 0.4 ^d^
*S. calva*	19.7 ± 0.1 ^c^	30.2 ± 0.2 ^d^	35.4 ± 0.2 ^d^

^a^ Sun protective factor. Results indicate ^b^ low UV protection; ^c^ medium UV protection; ^d^ high UV protection. Results were expressed as the mean ± standard error of the mean from three independent assays.

**Table 5:** Results of the antifungal activity of ethanolic extracts from *Sloanea* species.

Extract	Anti-*Candida* Activity, GM-MIC ^a^ (µg/mL)				
	*C. krusei* ATCC 6258 (L6)	*C. auris* Ca17 (L25)	*C. tropicalis* ATCC 200956	*C. glabrata* LMDM 34 (L7)	*C. albicans* ATCC 10231
*S. medusula*	32.0	16.0	32.0	22.6	2.0
*S. calva*	2.0	>256	4.0	>256	2.0
Itraconazole	0.125	-	-	-	-
Amphotericin B	0.630	-	-	-	-

^a^ the geometric mean of the minimum inhibitory concentration.

**Table 6:** Results of DPPH assays of extract-based gels.

Extract-Based Gel	DPPH ^a^ Radical Scavenging Activity (%) MEAN ± SEM (*n* = 3)	TEAC ^b^ MEAN ± SEM (*n* = 3)
*S. medusula* gel	54.5 ± 0.0	22.9 ± 0.0
*S. calva* gel	53.0 ± 0.0	20.3 ± 0.0

^a^ DPPH (1,1-diphenyl-2-picrylhydrazyl). ^b^ TEAC (Trolox equivalent antioxidant capacity). Results were expressed as the mean ± standard error of the mean from three independent assays.

**Table 7:** Results of determination of sun protective factor of *Sloanea*-extract-based gels.

Formulation	SPF ^a^ MEAN ± SEM (*n* = 3)
*S. medusula* gel	60.0 ± 0.0 ^b^
*S. calva* gel	57.3 ± 0.4 ^b^
*Sunscreen* 1 ^f^	60.0 ± 0.0 ^b^
*Sunscreen* 2 ^f^	47.1 ± 0.0 ^c^
Base gel	21.8 ± 0.6 ^d^

^a^ Sun protective factor. ^b^ Maximum UV protection. ^c^ High UV protection. ^d^ Good UV protection. ^f^ A commercial product that is marketed as having SPF 50. Results were expressed as the mean ± standard error of the mean from three independent assays.

**Table 8:** Results of the evaluation of the anti-*Candida albicans* activity of *Sloanea*-extract-based gels.

Gel, Final Concentration Evaluated (%)	GM-CFU/mL ^a^	Growth Inhibition (%)
*S. medusula* gel (0.15)	1260 *	98
*S. medusula* gel (0.30)	18,110 *	73
*S. calva* gel (0.15)	36,617 *	46
*S. calva* gel (0.30)	16,080 *	76
Base gel ^b^	67,824	0

^a^ Data are the geometric mean of colony-forming units per milliliter (*n* = 3). ^b^ The base gel was used as a positive control (growth control of *C. albicans*). The comparisons of results between the different groups were performed statistically using the paired *t*-test. * Significant difference in inhibitory activity when compared to the control group (*p* < 0.05).

**Table 9:** EE(λ) × I(λ) constant values to wavelength determinate.

Wavelength (λ, nm)	EE (λ) × I (λ)
290	0.015
295	0.082
300	0.287
305	0.328
310	0.186
315	0.084
320	0.018

**Table 10:** Composition of gels.

Composition of Formulation, Concentration	*S. medusula* Gel	*S. calva* Gel
Carbopol^®^ Ultrez 21 Polymer, % *w*/*w*	0.46	0.7
Sodium nipagin, % *w*/*w*	0.2	0.2
Triethanolamine, % *w*/*v*	1.0	1.0
Glycerine, % *w*/*v*	6.0	6.0
Deionized water, q.s.p., % *w*/*v*	100	100
Extract of *Sloanea* species, % *w*/*w*	0.3	0.3

## Data Availability

Data is contained within the article and supplementary material.

## Supplementary Materials

The following supporting information can be downloaded at: https://www.mdpi.com/article/10.3390/ph16070990/s1, Figure S1: Chromatograms of ethanolic extracts. (A) *Sloanea medusula* in negative ion mode, (B) *S. medusula* in positive ion mode, (C) *S. calva* in negative ion mode, and (D) *S. calva* in positive ion mode; Table S1: Proposed fragments and proposed neutral losses for the tentative annotations identified in ethanolic extracts from *S. medusula* and *S. calva*; Figure S2: 2D chemical structure (drawn in black color), proposed fragments (drawn in blue color), proposed neutral losses (drawn in red color), and MS-MS spectrum for the tentative annotation glycolic acid 4-hydroxy-3,5-di-t-butylbenzyl ester; Figure S3: 2D chemical structure (drawn in black color), proposed fragments (drawn in blue color), proposed neutral losses (drawn in red color), and MS-MS spectrum for the tentative annotation α-sorinin; Figure S4: 2D chemical structure (drawn in black color), proposed fragments (drawn in blue color), proposed neutral losses (drawn in red color), and MS-MS spectrum for the tentative annotation geraniin; Figure S5: 2D chemical structure (drawn in black color), proposed fragments (drawn in blue color), proposed neutral losses (drawn in red color), and MS-MS spectrum for the tentative annotation granatin B; Figure S6: 2D chemical structure (drawn in black color), proposed fragments (drawn in blue color), proposed neutral losses (drawn in red color), and MS-MS spectrum for the tentative annotation uralenic acid; Figure S7: 2D chemical structure (drawn in black color), proposed fragments (drawn in blue color), proposed neutral losses (drawn in red color), and MS-MS spectrum for the tentative annotation asiatic acid; Figure S8: 2D chemical structure (drawn in black color), proposed fragments (drawn in blue color), proposed neutral losses (drawn in red color), and MS-MS spectrum for the tentative annotation asiatic acid triacetate; Table S2: *In vitro* photoprotective activity measurement of ethanolic extracts and extract-based gels of *Sloanea medusula* and *S. calva;* Figure S9: Experimental design for evaluating the anti-*Candida albicans* activity of gels based on *S. medusula* and *S. calva*.
