# Supplementary material for: Exploring the Potential of Extracts from Sloanea medusula and S. calva: Formulating Two Skincare Gels with Antioxidant, Sun Protective Factor, and Anti-Candida albicans Activities
Source: Pharmaceuticals (Basel). 2023 Jul 11;16(7):990. doi: 10.3390/ph16070990 (PMC10384365; doi:10.3390/ph16070990)
Supplement: Supplementary file 1 [file pharmaceuticals-16-00990-s001.zip › pharmaceuticals-2472141-supplementary.pdf]

SUPPLEMENTARY MATERIAL

Exploring the Potential of Extracts from *Sloanea medusula* and *S. calva*: Formulating Two Skincare Gels with Antioxidant, Sun Protective Factor, and Anti-*Candida albicans* Activities

Patricia Quintero-Rincón[1,2], Ana C. Mesa-Arango[3], Oscar A. Flórez-Acosta[2], Carolina Zapata-Zapata[3], Elena E. Stashenko[4] and Nayive Pino-Benítez[1,*]

[1]Natural Products Group, Technological University of Chocó, Quibdó, Chocó, Colombia; nayivepino@gmail.com (N P-B); patriciaquintero@gmail.com (P Q-R).

[2]Research Group Design and Formulation of Medicines, Cosmetics, and Related, Faculty of Pharmaceutical and Food Sciences, University of Antioquia, Medellín-Colombia; oscar.florez@udea.edu.co (OA. F-A); (P Q-R).

[3]Academic Group of Clinical Epidemiology, Faculty of Medicine, University of Antioquia, Medellín-Colombia; ana.mesa@udea.edu.co (AC. M-A); carolina.zapataz@udea.edu.co (C Z-Z).

[4]Center for Chromatography and Mass Spectrometry, CROM-MASS, CIBIMOL-CENIVAM, Industrial University of Santander, Bucaramanga, Colombia; elena@tucan.uis.edu.co (EE. S).

***Correspondence**: nayivepino@gmail.com; +57 310 4559617.

CONTENT

Figure S1: Chromatograms of ethanolic extracts. (A) *Sloanea medusula* in negative ion mode, (B) *S. medusula* in positive ion mode, (C) *S. calva* in negative ion mode, and (D) *S. calva* in positive ion mode.

Table S1: Proposed fragments and proposed neutral losses for the tentative annotations identified in ethanolic extracts from *S. medusula* and *S. calva*.

Figure S2: 2D chemical structure (drawn in black color), proposed fragments (drawn in blue color), proposed neutral losses (drawn in red color), and MS-MS spectrum for the tentative annotation glycolic acid 4-hydroxy-3,5-di-t-butylbenzyl ester.

Figure S3: 2D chemical structure (drawn in black color), proposed fragments (drawn in blue color), proposed neutral losses (drawn in red color), and MS-MS spectrum for the tentative annotation α-sorinin.

Figure S4: 2D chemical structure (drawn in black color), proposed fragments (drawn in blue color), proposed neutral losses (drawn in red color), and MS-MS spectrum for the tentative annotation geraniin.

Figure S5: 2D chemical structure (drawn in black color), proposed fragments (drawn in blue color), proposed neutral losses (drawn in red color), and MS-MS spectrum for the tentative annotation granatin B.

Figure S6: 2D chemical structure (drawn in black color), proposed fragments (drawn in blue color), proposed neutral losses (drawn in red color), and MS-MS spectrum for the tentative annotation uralenic acid.

Figure S7: 2D chemical structure (drawn in black color), proposed fragments (drawn in blue color), proposed neutral losses (drawn in red color), and MS-MS spectrum for the tentative annotation asiatic acid.

Figure S8: 2D chemical structure (drawn in black color), proposed fragments (drawn in blue color), proposed neutral losses (drawn in red color), and MS-MS spectrum for the tentative annotation asiatic acid triacetate.

Table S2: *In vitro* photoprotective activity measurement of ethanolic extracts and extract-based gels of *Sloanea medusula* and *S. calva.*

Figure S9: Experimental design for evaluating the anti-*Candida albicans* activity of gels based on *S. medusula* and *S. calva*.

References

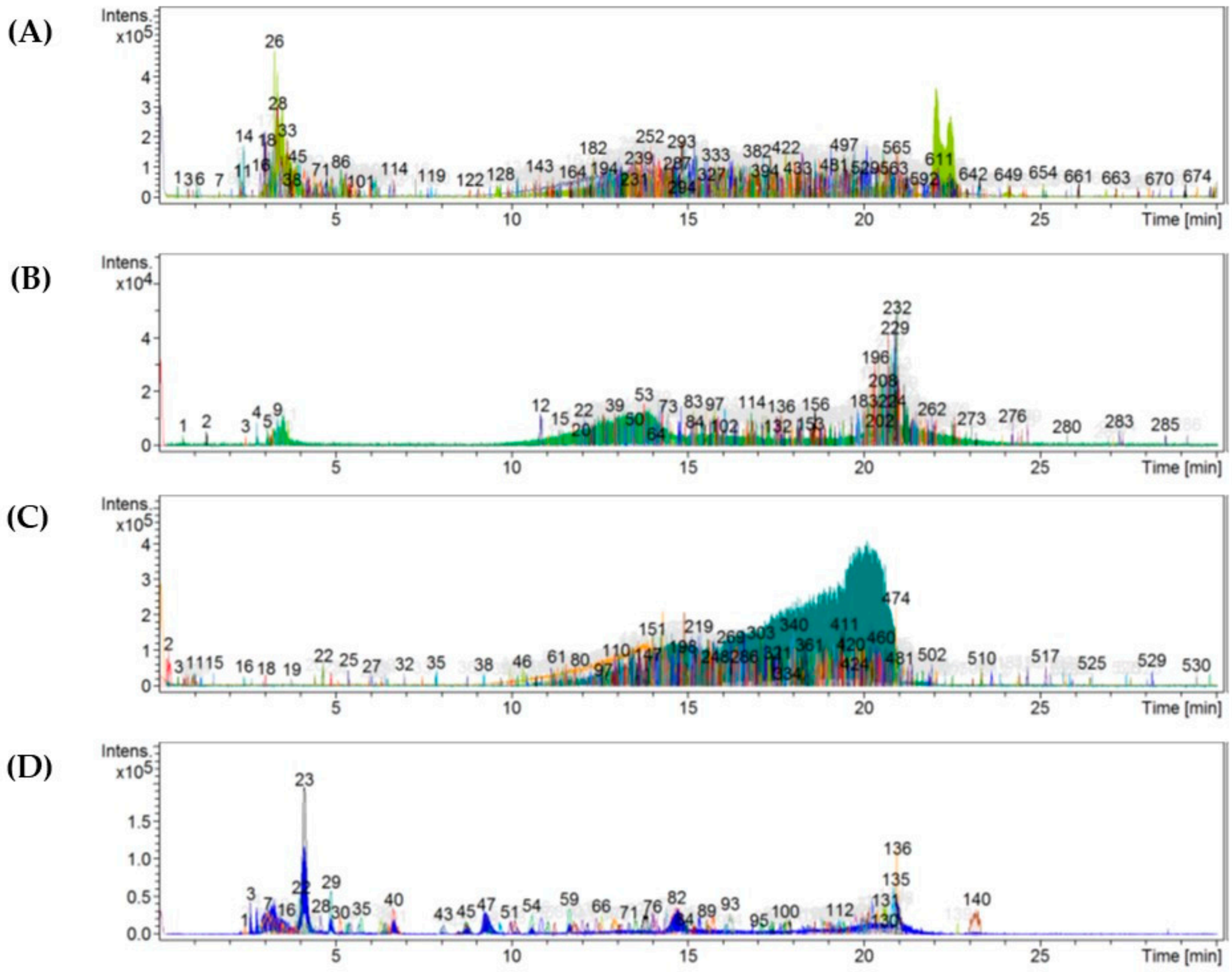

Figure S1: Chromatograms of ethanolic extracts. (A) *Sloanea medusula* in negative ion mode, (B) *S. medusula* in positive ion mode, (C) *S. calva* in negative ion mode, and (D) *S. calva* in positive ion mode.

Table S1: Proposed fragments and proposed neutral losses for the tentative annotations identified in ethanolic extracts from *S. medusula* and *S. calva*.

Tentative annotation	Structural Formula	Fragment ion peaks, *m/z* (intensity, %)	Proposed fragments	Neutral loss fragments (Da)	Proposed neutral loss	Fragment ion peaks from literature reports
Glycolic acid 4-hydroxy-3,5-di-t-butylbenzyl ester	$C_{17}H_{26}O_4$	219 (94)	$C_{15}H_{23}O$	76	$C_2H_4O_3$	Not Found
		236 (100)	$C_{13}H_{17}O_4$	58	C_4H_{10}	
α-Sorinin	$C_{24}H_{28}O_{14}$	227 (37)	$C_{13}H_7O_4$	314	$C_5H_{10}O_4$, $C_6H_{12}O_6$	Not Found
		313 (100)	$C_{11}H_{21}O_{10}$	226	$C_{13}H_6O_4$	
		387 (89)	$C_{19}H_{15}O_9$	152	$C_5H_{10}O_4$, H_2O	
Geraniin	$C_{41}H_{28}O_{27}$	169 (100)	$C_7H_5O_5$	782	$C_{34}H_{22}O_{22}$	ESI-MS, positive mode: *m/z* 951, 554, 446, 247 [41]
		300 (80)	$C_{14}H_5O_8$	651	$C_{27}H_{23}O_{19}$	
		431 (66)	$C_{20}H_{15}O_{11}$	520	$C_{21}H_{13}O_{16}$	
Granatin B	$C_{41}H_{28}O_{27}$	169 (100)	$C_7H_5O_5$	782	$C_{34}H_{22}O_{22}$	ESI-MS, negative mode: *m/z* 951, 783, 605, 300 [42]
		300 (80)	$C_{14}H_5O_8$	651	$C_{27}H_{23}O_{19}$	
		431 (66)	$C_{20}H_{15}O_{11}$	520	$C_{21}H_{13}O_{16}$	
Uralenic acid, glycyrrhetinic acid	$C_{30}H_{46}O_4$	205 (28.2)	$C_{15}H_{25}$	266	$C_{15}H_{22}O_3$, H_2O	ESI-MS, positive mode: *m/z* 493, 471, 177 [44]
		219 (32.4)	$C_{15}H_{23}O$	252	$C_{15}H_{23}O_3$	
		223 (44.7)	$C_{14}H_{23}O_2$	248	$C_{16}H_{24}O_2$	
		407 (38.5)	$C_{29}H_{43}O$	64	HCOOH, H_2O	
		425 (100)	$C_{29}H_{44}O_2$	46	HCOOH	
Asiatic acid	$C_{30}H_{48}O_5$	187 (28.5)	$C_{10}H_{35}O_2$	302	$C_{20}H_{14}O_3$	ESI-MS, negative mode: *m/z* 487 [46]
		205 (100)	$C_{15}H_{25}$	284	$C_{13}H_{16}O_2$, 2(CH_3OH), H_2O	ESI-MS, negative mode: *m/z* 688, 455, 365 [47]
		223 (19.4)	$C_{14}H_{23}O_2$	266	$C_{16}H_{26}O_3$	
		407 (44.6)	$C_{29}H_{43}O$	82	HCOOH, 2(H_2O)	
		425 (77)	$C_{29}H_{45}O_2$	64	HCOOH, H_2O	ESI-MS, negative mode: *m/z* 409 [48]
		453 (18.7)	$C_{30}H_{45}O_3$	36	2(H_2O)	
Asiatic acid triacetate	$C_{36}H_{54}O_8$	219 (33.5)	$C_{16}H_{27}$	396	$C_{20}H_{28}O_8$	ESI-MS, negative mode: *m/z* 628, 612, 605 [47]
		407 (100)	$C_{29}H_{43}O$	206	3($C_2H_4O_2$), C_2H_4	
		453 (20)	$C_{31}H_{49}O_2$	162	2($C_2H_4O_2$), C_3H_6	

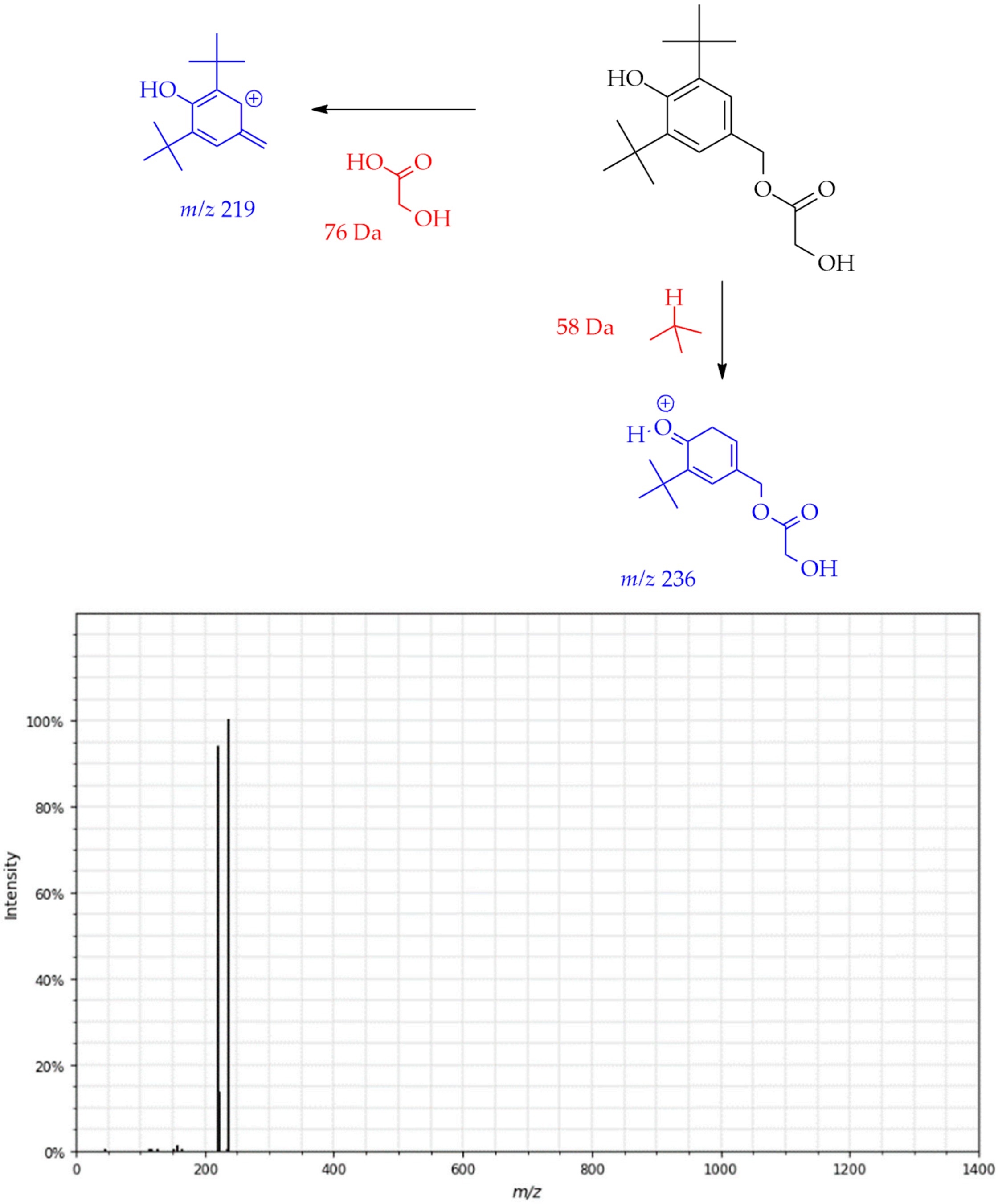

Figure S2: 2D chemical structure (drawn in black color), proposed fragments (drawn in blue color), proposed neutral losses (drawn in red color), and MS-MS spectrum for the tentative annotation glycolic acid 4-hydroxy-3,5-di-t-butylbenzyl ester.

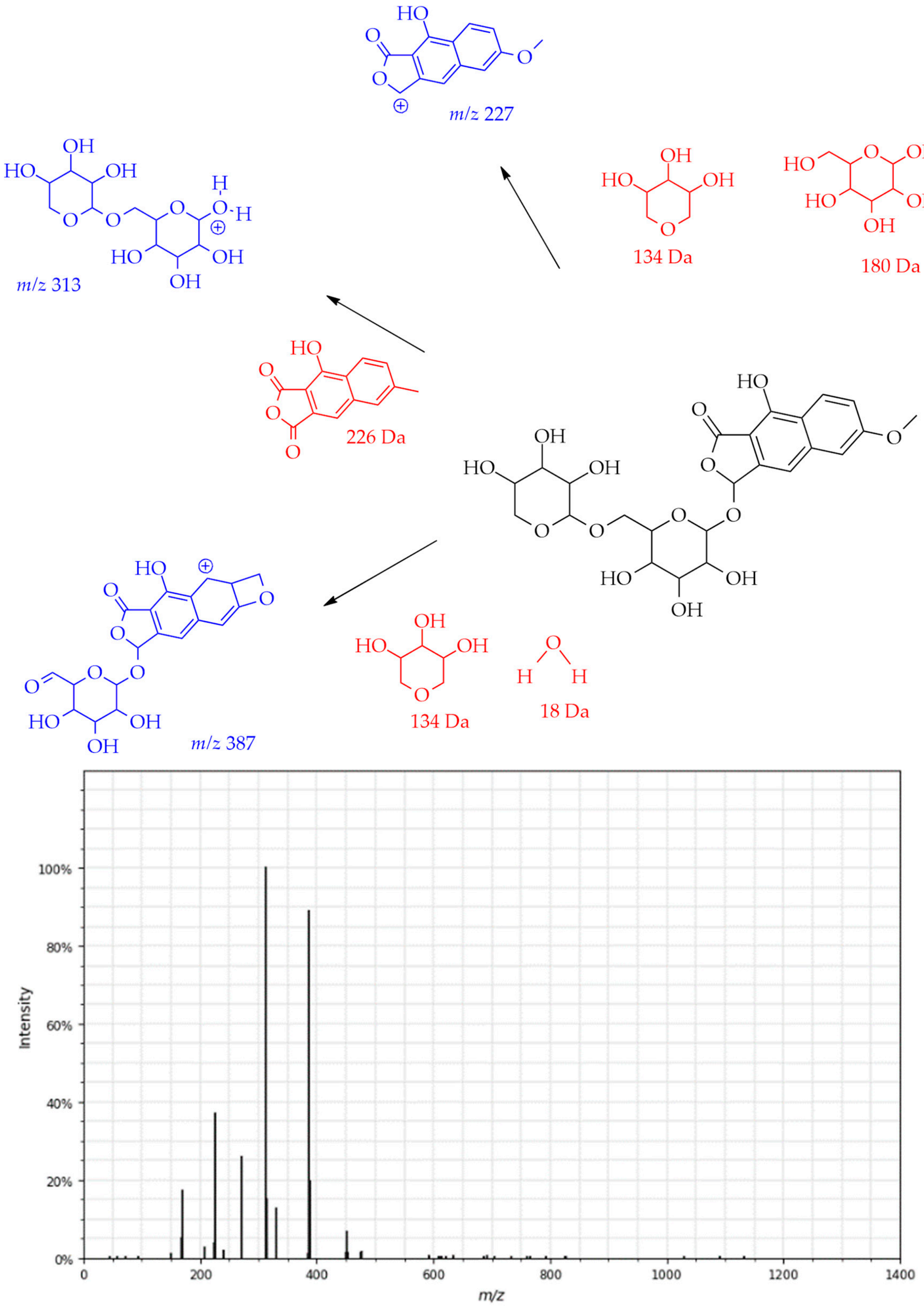

Figure S3: 2D chemical structure (drawn in black color), proposed fragments (drawn in blue color), proposed neutral losses (drawn in red color), and MS-MS spectrum for the tentative annotation α-sorinin.

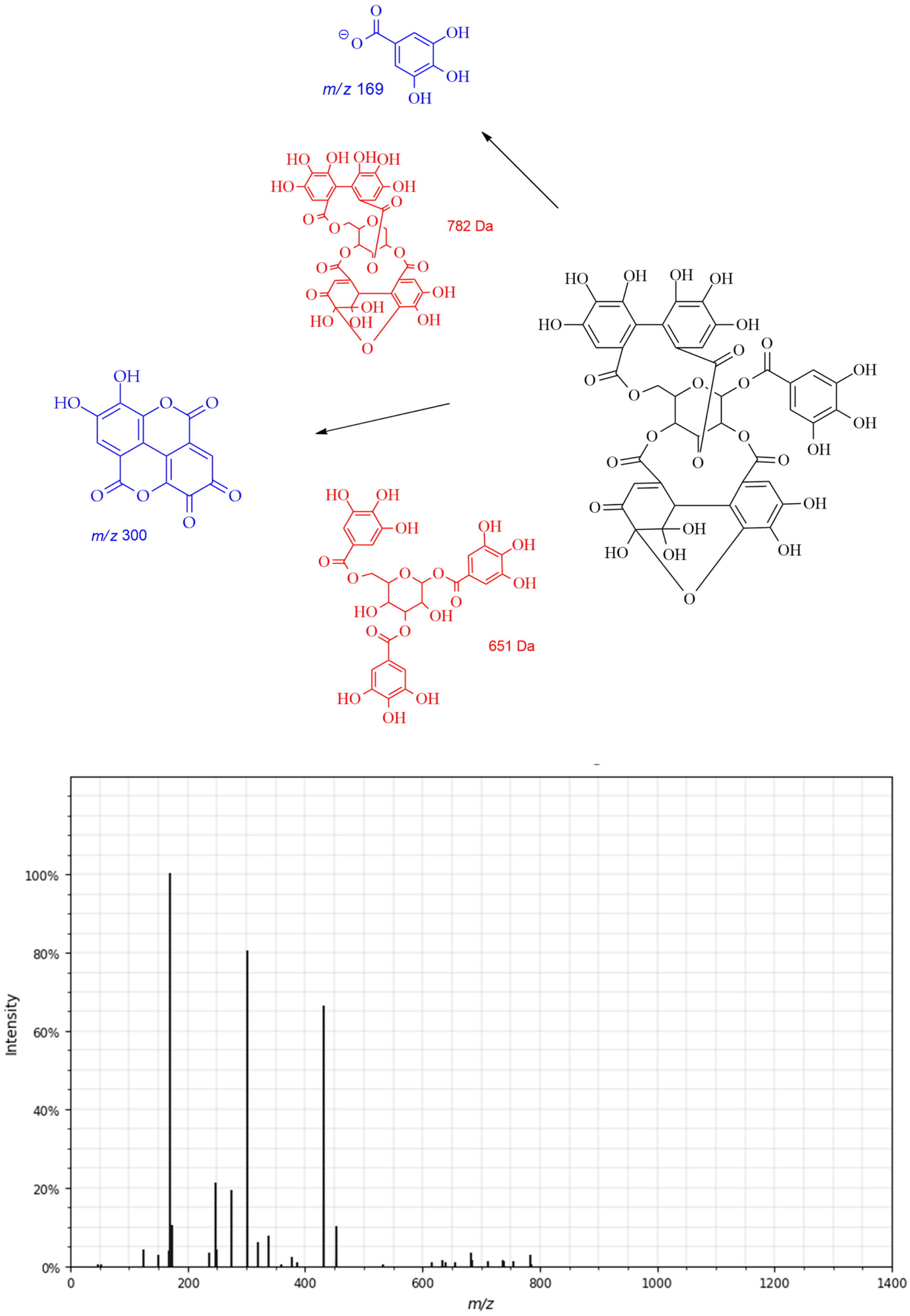

Figure S4: 2D chemical structure (drawn in black color), proposed fragments (drawn in blue color), proposed neutral losses (drawn in red color), and MS-MS spectrum for the tentative annotation geraniin.

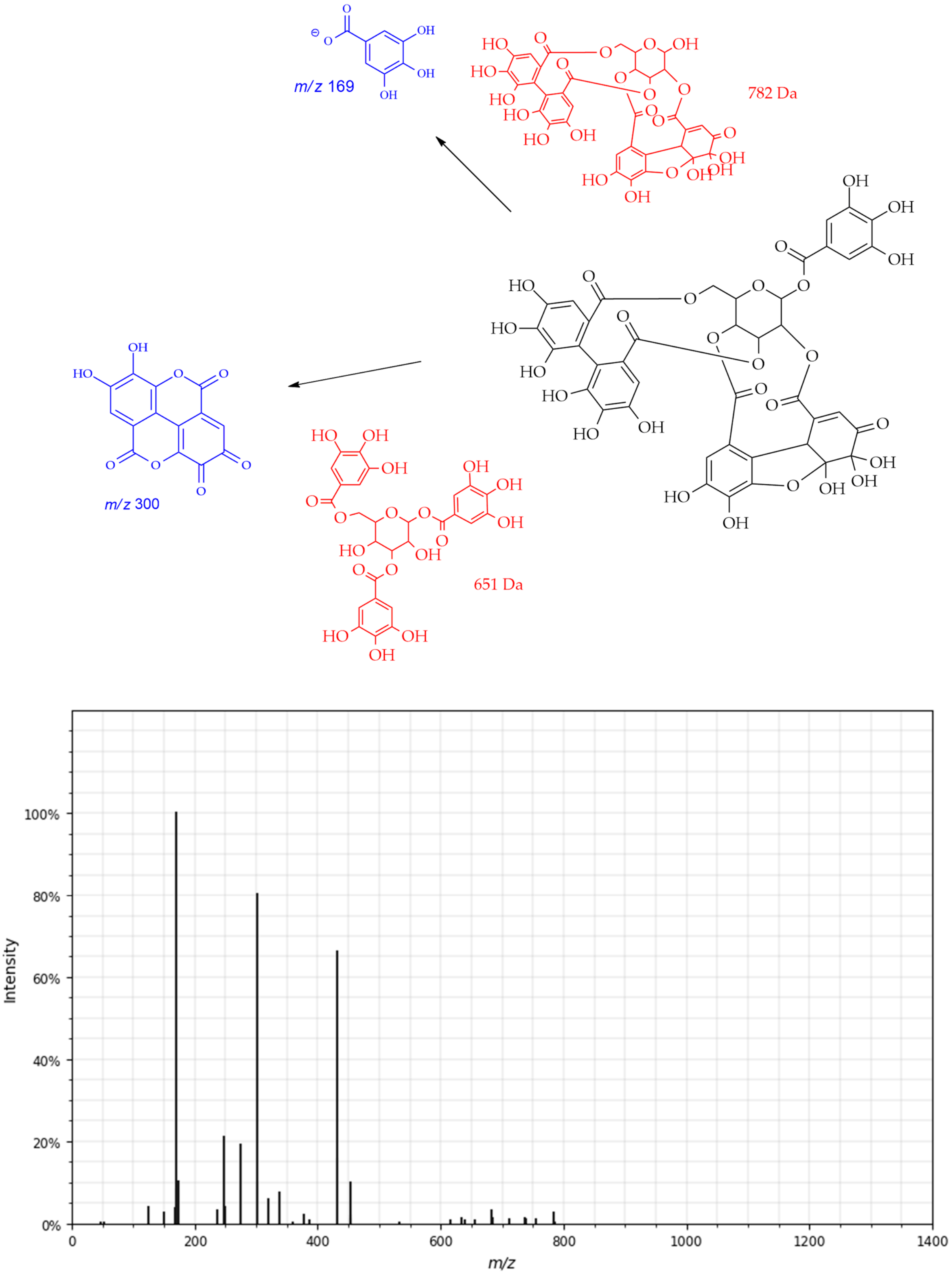

Figure S5: 2D chemical structure (drawn in black color), proposed fragments (drawn in blue color), proposed neutral losses (drawn in red color), and MS-MS spectrum for the tentative annotation granatin B.

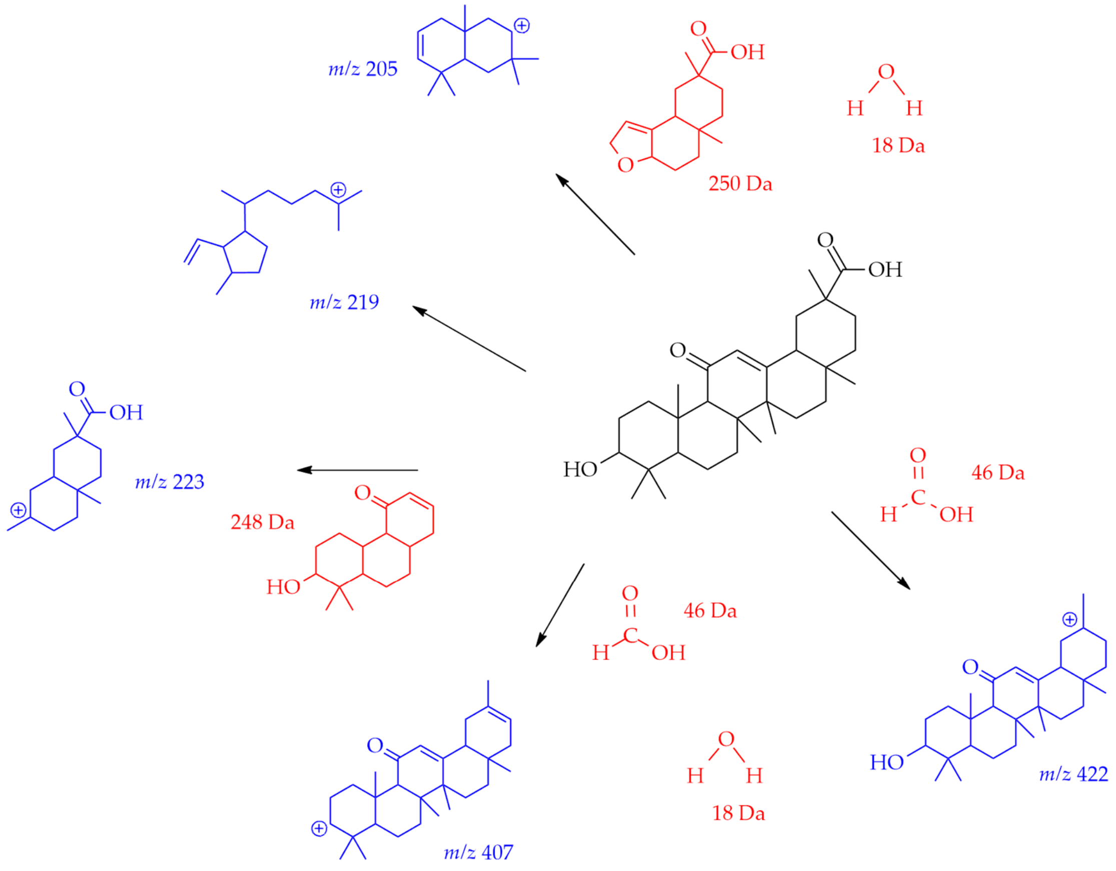

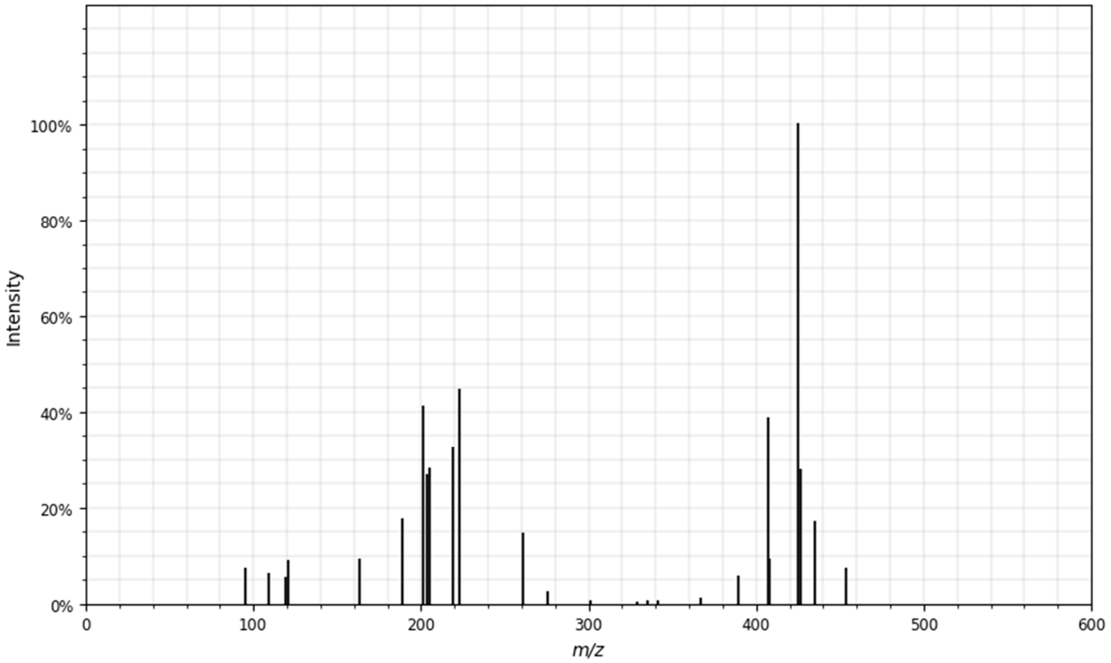

Figure S6: 2D chemical structure (drawn in black color), proposed fragments (drawn in blue color), proposed neutral losses (drawn in red color), and MS-MS spectrum for the tentative annotation uralenic acid.

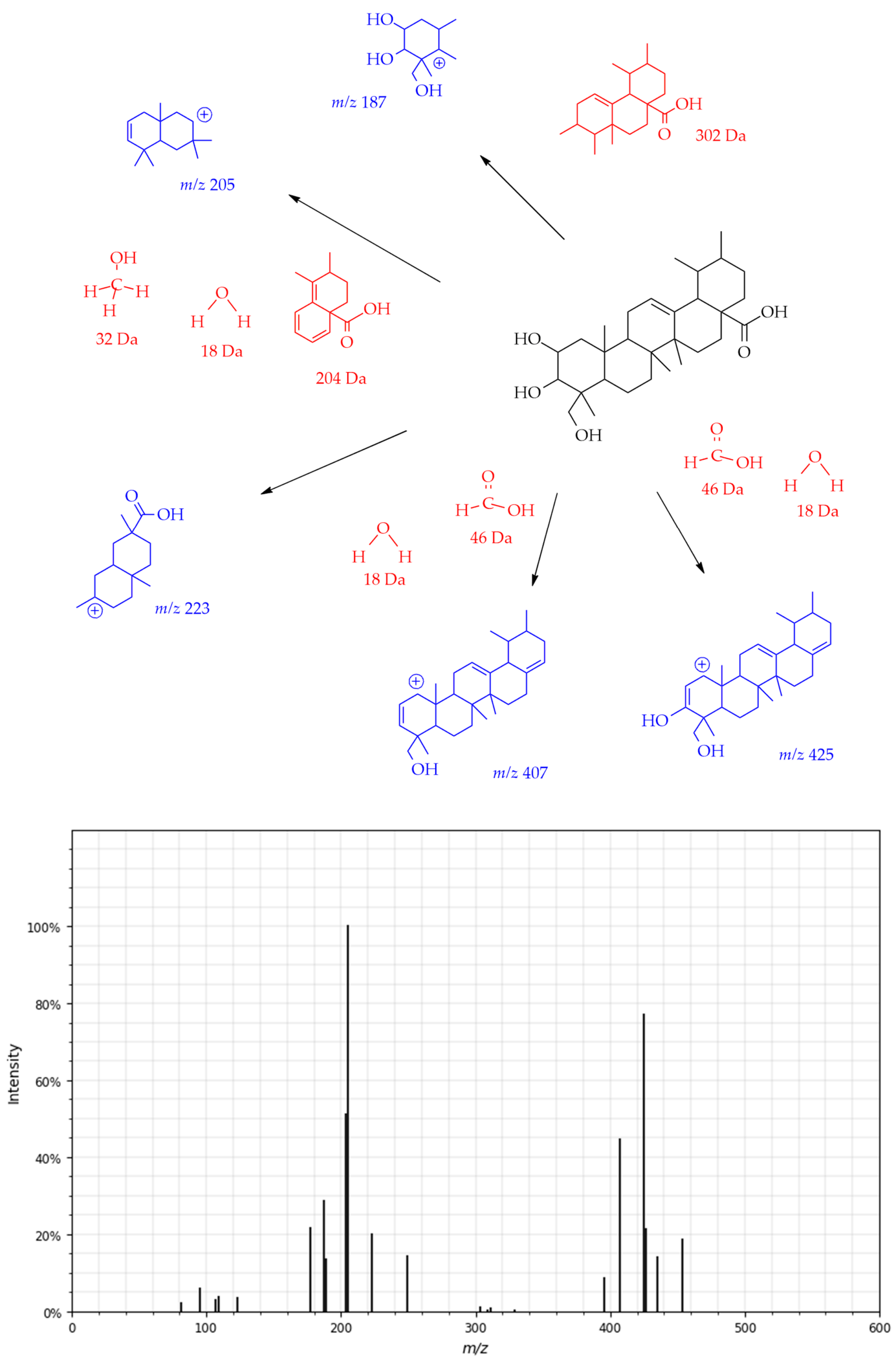

Figure S7: 2D chemical structure (drawn in black color), proposed fragments (drawn in blue color), proposed neutral losses (drawn in red color), and MS-MS spectrum for the tentative annotation asiatic acid.

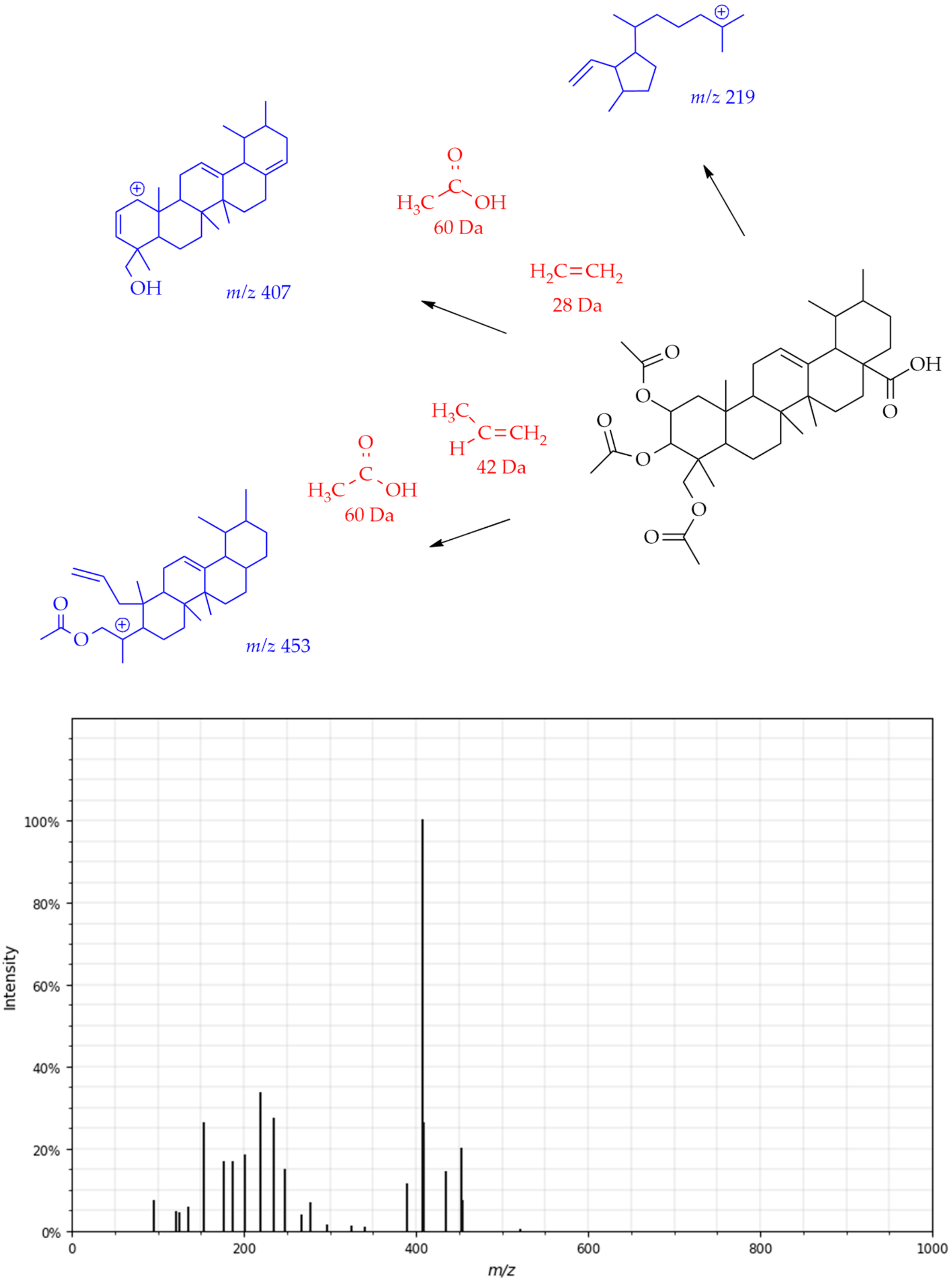

Figure S8: 2D chemical structure (drawn in black color), proposed fragments (drawn in blue color), proposed neutral losses (drawn in red color), and MS-MS spectrum for the tentative annotation asiatic acid triacetate.

Table S2: *In vitro* photoprotective activity measurement of ethanolic extracts and extract-based gels of *Sloanea medusula* and *S. calva.*

Extract	Concentration (mg/mL)	UVA/UVB ratio	Transmission of erythema (%)	Transmission of pigmentation (%)
Sloanea medusula	0.25	0.7 ± 0.0	0.6 ± 0.0	23.0 ± 0.1
	0.5	0.8 ± 0.0	0.1 ± 0.0	11.1 ± 0.6
	0.75	0.9 ± 0.0	0.0 ± 0.0	6.0 ± 0.3
S. calva	0.25	0.7 ± 0.0	0.3 ± 0.0	14.9 ± 0.1
	0.5	0.9 ± 0.0	0.0 ± 0.0	5.4 ± 0.1
	0.75	1.1 ± 0.0	0.0 ± 0.0	1.9 ± 0.1
**Extract-based gel or sunscreen**		**UVA/UVB ratio**	**Transmission of erythema (%)**	**Transmission of pigmentation (%)**
***S. medusula* gel (0.30 %)** [a]		1.8 ± 0.1	0.0 ± 0.0	0.0 ± 0.0
***S. calva* gel (0.30 %)** [a]		1.9 ± 0.1	0.0 ± 0.0	0.0 ± 0.0
**Base gel** [a]		0.1 ± 0.0	0.2 ± 0.0	33.6 ± 0.7
***Sunscreen* 1** [a]		2.6 ± 0.0	0.0 ± 0.0	0.0 ± 0.0
***Sunscreen* 2** [a]		2.4 ± 0.2	0.0 ± 0.0	0.0 ± 0.0

Results are MEAN ± SEM (n = 3). The star rating system indicates that the UVA/UVB ratio of 0.6 to <0.8 is a superior protector (***), and 0.8 to ≥0.8 is a maximum protector (****). Values of the transmission of erythema (< 1%) and the transmission of pigmentation (0-40%) indicate the effectiveness of extracts and gels as sunscreen in preventing the appearance of skin redness and dark spots after exposure to UV radiation, respectively. These values should be as low as possible to their consideration as sunscreen. [a]The photoprotective activity measurement was obtained with a mixture of gel (or sunscreen) and ethanol in a 1:1 ratio. Parameters were evaluated following the methodology employed by Caballero-Gallardo et al. [29].

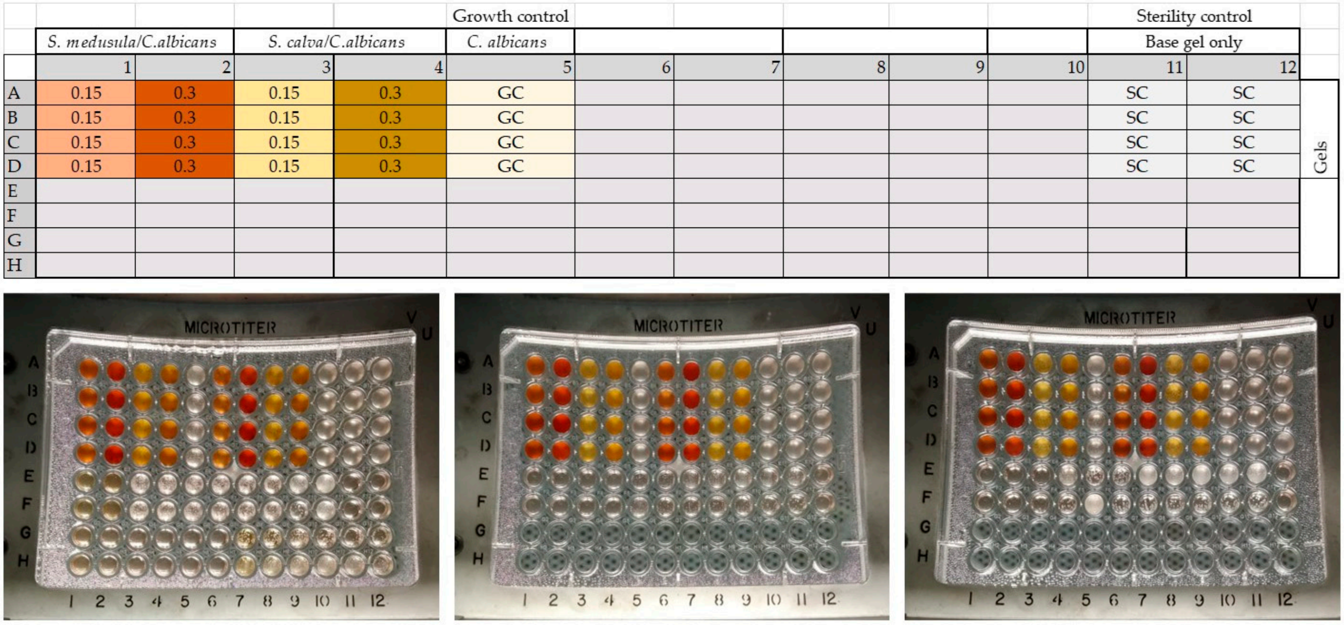

All assays were developed using the CLSI Standard M27, 4th Edition with minor modifications [80]. The plates were incubated at 35°C. After 24 h of incubation, they were used to perform colony-forming unit experiments and quantify the percentage of growth inhibition of *Candida albicans* in CFU/mL. No microorganism growth was observed in the sterility control. These assays were conducted on different days.

Figure S9: Experimental design for evaluating the anti-*Candida albicans* activity of gels based on *S. medusula* and *S. calva*.
